# Enhanced dispersion stability of gold nanoparticles by the physisorption of cyclic poly(ethylene glycol)

**DOI:** 10.1038/s41467-020-19947-8

**Published:** 2020-11-30

**Authors:** Yubo Wang, Jose Enrico Q. Quinsaat, Tomoko Ono, Masatoshi Maeki, Manabu Tokeshi, Takuya Isono, Kenji Tajima, Toshifumi Satoh, Shin-ichiro Sato, Yutaka Miura, Takuya Yamamoto

**Affiliations:** 1 Graduate School of Chemical Sciences and Engineering, Hokkaido University, Sapporo, Hokkaido 060-8628 Japan; 2 Division of Applied Chemistry, Faculty of Engineering, Hokkaido University, Sapporo, Hokkaido 060-8628 Japan; 3 Department of Bioengineering, Graduate School of Engineering, The University of Tokyo, 7-3-1 Hongo, Bunkyo-ku, Tokyo 113-8656 Japan; 4 Laboratory for Chemistry and Life Science, Institute of Innovative Research, Tokyo Institute of Technology, 4259 Nagatsutacho, Midori-ku, Yokohama, Kanagawa 226-8503 Japan

**Keywords:** Polymer chemistry, Biopolymers, Nanobiotechnology, Nanoparticles

## Abstract

Nano-sized metal particles are attracting much interest in industrial and biomedical applications due to the recent progress and development of nanotechnology, and the surface-modifications by appropriate polymers are key techniques to stably express their characteristics. Herein, we applied cyclic poly(ethylene glycol) (*c*-PEG), having no chemical inhomogeneity, to provide a polymer topology-dependent stabilization for the surface-modification of gold nanoparticles (AuNPs) through physisorption. By simply mixing *c*-PEG, but not linear counterparts, enables AuNPs to maintain dispersibility through freezing, lyophilization, or heating. Surprisingly, *c*-PEG endowed AuNPs with even better dispersion stability than thiolated PEG (HS–PEG–OMe). The stronger affinity of *c*-PEG was confirmed by DLS, ζ-potential, and FT-IR. Furthermore, the *c*-PEG system exhibited prolonged blood circulation and enhanced tumor accumulation in mice. Our data suggests that *c*-PEG induces physisorption on AuNPs, supplying sufficient stability toward bio-medical applications, and would be an alternative approach to the gold–sulfur chemisorption.

## Abstract (web summary)

Many nanoparticles are stabilised by chemical functionalisation with poly(ethylene glycol) (PEG). Here, the authors report on the functionalisation of gold nanoparticles by the physical absorption of cyclic PEG and demonstrate superior stabilisation against heating, freezing and lyophilisation.

## Introduction

Metal nanoparticles represent a widely interesting group of materials due to their unique properties which differ from their respective bulk state. Presently, metal nanoparticles are used in a wide range of applications which include optics^1^, sensors^2^, biomedicine^3^, electronics^4^, and catalysis^5^. Metal nanoparticles such as gold, silver, and copper nanoparticles exhibit surface plasmon resonances (SPR) as a result of their interaction with light which manifests itself in the different colors of the corresponding colloidal solutions depending on the particle size and shape^6^. However, metal nanoparticles are not a stable material and easily aggregate to lose SPR absorption and thus need to be kept as a solution at low temperature. Yet, freezing the solution also causes aggregation and cannot be redispersed. Moreover, the dispersion stability in solution is also affected by various parameters such as salt concentration^7^, temperature^8^ or pH^9^, all of which eventually lead to particle dissolution or aggregation. Hence, metal nanoparticles are often either kept in a citrate solution or reacted with thiolated molecules to gain dispersion stability^10^.

Over the past decades, there have been numerous reports on the dispersion stabilization of gold nanoparticles (AuNPs), the most representative metal nanoparticles, using poly(ethylene glycol) (PEG) as a stabilizer, namely PEGylation^11^. PEG is a neutral water-soluble polymer with a flexible backbone and is well-known for its ability to bind to water through hydrogen bonding^12^. Additionally, PEG is biocompatible and can protect gold surfaces from aggregation in vitro and in vivo as well as from detection by the immune system^13^. When biological applications are considered, the stability against salts in physiological conditions is an indispensable property. The increase in the ionic strength through the addition of a salt or electrolyte reportedly leads to aggregation^13^. Therefore, proper surface-modification is essential to avoid aggregation. However, PEGylation, as well as other functionalizations, of AuNPs has been almost limited to chemisorption by the gold–sulfur reactions, and thus, it is important to establish a new PEGylation methodology for the development of nanoparticle sciences.

Although the stabilization of nanoparticles by a polymer has extensively been explored over the years by tuning various parameters such as molecular weight^14^ or composition^15^, very little attention has been paid on the potential contribution of the polymer topology. Over the last decade, numerous articles on the cyclization of various polymers through different routes have been reported which yielded polymers with different topologies^16,17^. The cyclized polymers exhibited distinct properties compared to their linear counterparts of the same molecular weight such as higher density, higher glass transition temperature, smaller hydrodynamic volume, and lower viscosity^18,19^. In particular, the dispersion stability exhibited by the cyclic polymers is an interesting feature which could be exploited for the stability of nanoparticles. Thus, we reported that micelles composed of cyclized PEG-containing amphiphilic block copolymers feature a higher tolerance towards temperature and salinity compared to their linear counterparts in an aqueous solution, which was proven by turbidity tests and dynamic light scattering^20–22^. Moreover, cyclic polyoxazoline chemisorbed to a titanium oxide surface, Fe_3_O_4_ nanoparticles, or a degraded cartilage formed a denser layer than the linear counterpart^23–25^.

In this work, the use of PEG as a polymeric stabilizer of AuNPs, where the effects of the polymer topology (linear vs. cyclic) as well as the end groups (–OH vs. –OMe vs. –SH) against freezing, lyophilization, heating, and a physiological condition, is investigated with the above observations in mind (Supplementary Fig. 1). In consequence, we find that *c*-PEG endows AuNPs with enhanced dispersion stability.

## Results

### Synthesis of *c*-PEG

**Fig. 1:**
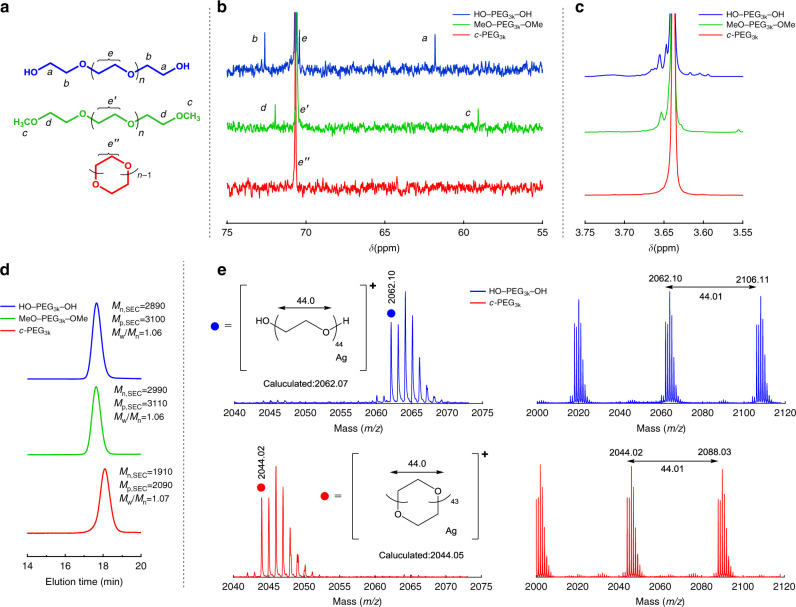
Characterisation data for HO–PEG_3k_–OH, MeO–PEG_3k_–OMe, and *c*-PEG_3k_. **a** Chemical structures, **b** ^13^C NMR spectra, **c** ^1^H NMR spectra, **d** SEC traces, and **e** MALDI-TOF mass spectra of HO–PEG_3k_–OH (blue), MeO–PEG_3k_–OMe (green), and *c*-PEG_3k_ (red).

PEG was cyclized via etherification, thus forming cyclic PEG (*c*-PEG) without inhomogeneity in the chemical structure, by using the modified tosylation method according to previous reports (Fig. 1a)^26,27^. The molecular weight was 1, 3, 5, and 10 kDa (namely, *c*-PEG_1k_, *c*-PEG_3k_, *c*-PEG_5k_, and *c*-PEG_10k_, respectively, shown in Table 1). The expected diameter of *c*-PEG_1k_, *c*-PEG_3k_, *c*-PEG_5k_, and *c*-PEG_10k_ was 2.2, 7.4, 14, and 28 nm, respectively, when they form an ideal right circular conformation. The shift in the size exclusion chromatography (SEC) traces confirms the change in the polymer topology as a result of the decrease in the hydrodynamic volume upon cyclization (Fig. 1d and Supplementary Fig. 2). For example, peak top molecular weight (*M*_p,SEC_) of PEG_3k_ decreased from 3100 to 2090 upon cyclization. The small values of polydispersity index (*M*_w_/*M*_n_), except for PEG_1k_, allowed for clear discussion on the molecular-weight dependence. Furthermore, matrix-assisted laser desorption ionization time-of-flight (MALDI-TOF) mass spectrometry and NMR also featured the difference between the linear and cyclic polymer species. For instance, the MALDI-TOF mass spectrum of HO‒PEG_3k_‒OH showed a peak at *m*/*z* = 2062.10 (DP_n_ = 44, Ag^+^ adduct), whereas the corresponding peak from *c*-PEG was at *m*/*z* = 2044.02; the value in the isotope distribution shifted by a mass unit of 18 due to the net elimination of a water molecule upon cyclization (Fig. 1e and Supplementary Fig. 3). The experimental *m*/*z* values for the monoisotopic mass of *c*-PEG_3k_ well matched to the calculated values for its molecular weight distribution range (Supplementary Table 1). In the ^13^C NMR spectra, the peak at 61.8 ppm and 72.5 ppm observed for the carbon atoms adjacent to the terminal hydroxy groups in HO‒PEG‒OH vanished upon cyclization, thus confirming the effective elimination of the end groups (Fig. 1b and Supplementary Fig. 4). ^1^H NMR also showed the high symmetry of *c*-PEG by the single peak of the methylene protons (Fig. 1c and Supplementary Fig. 5). MeO‒PEG‒OMe was synthesized from HO‒PEG‒OH with iodomethane under an alkaline condition^26^. Essentially, no absorption from purified PEG was confirmed by UV‒Vis spectroscopy, allowing for the clear observation of the SPR absorption of AuNPs. The comparison of highly pure *c*-PEG with HO‒PEG‒OH and MeO‒PEG‒OMe, along with HS‒PEG‒OMe and HS‒PEG‒SH, allowed for the stringent evaluation of the effects from the topology and the end groups.

**Table 1:** Properties of PEG by SEC.

Polymer	${\mathbf{M}}_{{\bf{n}},{\bf{SEC}}}^{\bf{a}}$ (g mol^−1^)	${\mathbf{M}}_{{\bf{p}},{\bf{SEC}}}^{\bf{a}}$ (g mol^−1^)	*M*_w_*/M*_n_	Ideal diameter of *c*-PEG (nm)
HO–PEG_1k_–OH	860	1220	1.35	—
*c*-PEG_1k_	430	520	1.38	2.2
HO–PEG_3k_–OH	2890	3100	1.06	—
MeO–PEG_3k_–OMe	2990	3110	1.06	—
HS–PEG_3k_–OMe	2500	2540	1.07	—
HS–PEG_2k_–SH	2220	2500	1.08	
*c*-PEG_3k_	1910	2090	1.07	7.4
HO–PEG_5k_–OH	5450	5480	1.03	—
MeO–PEG_5k_–OMe	5460	5480	1.03	—
*c*-PEG_5k_	3750	3860	1.05	14
HO–PEG_10k_–OH	10,800	11,060	1.01	—
MeO–PEG_10k_–OMe	11,760	12,140	1.01	—
*c*-PEG_10k_	8500	8810	1.02	28

^a^Determined by SEC in THF using polystyrene standards.

### Dispersion stability against freezing, lyophilization, heating, and a physiological condition

**Fig. 2:**
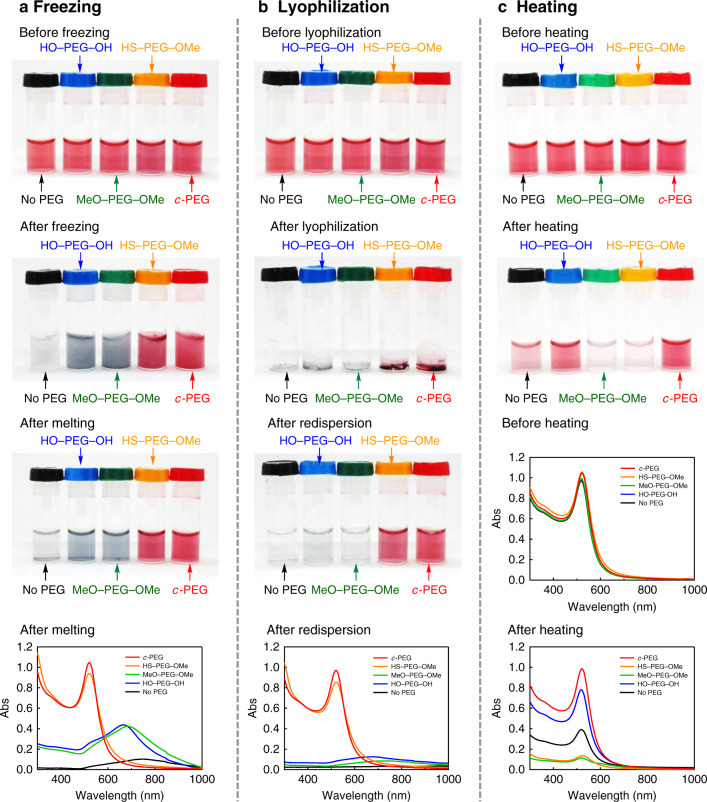
Freezing, lyophilization, and heating tests. Photographs and UV–Vis spectra of AuNPs_15_/No PEG (black), AuNPs_15_/HO–PEG_3k_–OH (blue), AuNPs_15_/MeO–PEG_3k_–OMe (green), AuNPs_15_/HS–PEG_3k_–OMe (orange), and AuNPs_15_/*c*-PEG_3k_ (red). **a** Freezing test using a household refrigerator. From top to bottom, photographs before freezing, after freezing, after melting, and a UV–Vis spectrum after melting. **b** Lyophilization test using liquid N_2_. From top to bottom, photographs before freezing, after lyophilization, after redispersion, and a UV–Vis spectrum after redispersion. The UV–Vis spectra before freezing and lyophilization are shown in Supplementary Fig. 6. **c** Heating test at 85 °C for 4 h. From top to bottom, photographs before and after heating and UV–Vis spectra before and after heating.

Based on the enhanced stability of the micelles form the cyclic block copolymers^20–22^, we tested *c*-PEG for the dispersion stabilization of AuNPs in comparison with the linear counterparts, finding significantly enhanced stability against temperature and change in the phase. Thus, HO–PEG_3k_–OH, MeO–PEG_3k_–OMe, HS–PEG_3k_–OMe, or *c*-PEG_3k_ was added to a commercially available aqueous dispersion of AuNPs with 15 nm in diameter (AuNPs_15_). By mixing AuNPs with these polymers, no change in the red color by the SPR absorption was observed. UV–Vis spectroscopy showed similar spectra to those before mixing with PEG, and *λ*_max_ remained at around 520 nm (Supplementary Fig. 6). Upon freezing in a household refrigerator, AuNPs_15_ without PEG (AuNPs_15_/No PEG) become nearly colorless due to aggregation^13^ and etching by dissolved oxygen (Fig. 2a)^28^. When AuNPs_15_ with HO–PEG_3k_–OH (AuNPs_15_/HO–PEG_3k_–OH) and with MeO–PEG_3k_–OMe (AuNPs_15_/MeO–PEG_3k_–OMe) were frozen, the red color of AuNPs disappeared and became grayish blue. After melting, UV–Vis spectra were recorded showing a strong bathochromic shift in *λ*_max_ (AuNPs_15_/HO–PEG_3k_–OH, 667 nm; AuNPs_15_/MeO–PEG_3k_–OMe, 687 nm), suggesting aggregation of AuNPs. The relative absorption intensity (Rel. Abs) compared to that before freezing was 26% or less for these three specimens listed in Table 2. In contrast, the red color of AuNPs_15_/HS–PEG_3k_–OMe (Rel. Abs = 92%) and AuNPs_15_/*c*-PEG_3k_ (Rel. Abs = 97%) persisted after melting with an unnoticeable change, and UV–Vis showed nearly identical spectra to those before freezing, demonstrating that the PEGylation by the two methods provide dispersibility through the frozen state.

**Table 2:** Relative absorption intensity (Rel. Abs) of AuNPs_15_/PEG_3k_ in freezing, lyophilization, heating, and physiological condition tests.

Test	No PEG	HO–PEG–OH	MeO–PEG–OMe	HS–PEG–OMe	*c*-PEG
Freezing	3%	26%	21%	92%	97%
Lyophilization	3%	9%	5%	84%	91%
Heating	39%	78%	12%	13%	95%
Physiological condition	1%	1%	2%	83%	88%

Rel. Abs was calculated by dividing the absorption value at *λ*_max_ after the tests by that before the tests.

Based on the above results, the effects of lyophilization of the dispersions were evaluated (Fig. 2b). In this experiment, the dispersions were frozen in liquid nitrogen, and the solvent was sublimed under reduced pressure. Dried AuNPs_15_/HS–PEG_3k_–OMe and AuNPs_15_/*c*-PEG_3k_ kept the red color, suggesting that AuNPs did not aggregate even in the solid state likely by the respective PEG molecules penetrate among AuNPs. Upon addition of water, AuNPs_15_/HS–PEG_3k_–OMe (Rel. Abs = 84%) and AuNPs_15_/*c*-PEG_3k_ (Rel. Abs = 91%) gave a redispersion with good restoration of the original spectra. On the other hand, AuNPs_15_/No PEG, AuNPs_15_/HO–PEG_3k_–OH, and AuNPs_15_/MeO–PEG_3k_–OMe formed grayish blue solid, and redispersion was not possible (Rel. Abs ≤ 9%). These experiments indicate that the physisorption of *c*-PEG can endow AuNPs with dispersion stability against freezing and lyophilization as good as the chemisorption of HS–PEG–OMe.

Furthermore, AuNPs_15_/No PEG, AuNPs_15_/HO–PEG_3k_–OH, AuNPs_15_/MeO–PEG_3k_–OMe, AuNPs_15_/HS–PEG_3k_–OMe, and AuNPs_15_/*c*-PEG_3k_ were heated at 85 °C for 4 h to determine the thermal stability. Most of the nanoparticles in AuNPs_15_/MeO–PEG_3k_–OMe (Rel. Abs = 12%) and AuNPs_15_/HS–PEG_3k_–OMe (Rel. Abs = 13%) aggregated to deposit or etched by dissolved oxygen to give almost colorless liquid (Fig. 2c)^28^. AuNPs_15_/No PEG (Rel. Abs = 39%) and AuNPs_15_/HO–PEG_3k_–OH (Rel. Abs = 78%) showed some degree of retention of the red color but less intense than those before heating. Interestingly, the color and UV–Vis spectrum of AuNPs_15_/*c*-PEG_3k_ were nearly intact (Rel. Abs = 95%), suggesting excellent dispersion stability against heating.

In order to determine the cause of the relatively poor protection by HS–PEG_3k_–OMe against heating, ^1^H NMR was used to find that the thiol group turned into disulfide (–C*H*_2_–S–S–C*H*_2_–, 2.91 ppm) (Supplementary Fig. 7). ^13^C NMR before and after heating also suggests the formation of disulfide (Supplementary Fig. 8). It is known that the bonding of Au and HS–PEG is dynamic^29^, and we expected that at high temperature, HS–PEG_3k_–OMe is liberated from the surface of AuNPs and oxidized to form disulfide (MeO–PEG_3k_–S–S–PEG_3k_–OMe). Once disulfide is formed, the affinity to Au becomes much smaller than that of thiol^30^, leading to the less shielding on AuNPs. In the meantime, HO–PEG_3k_–OH showed moderate dispersion stability most likely by the effects of the hydroxy chain end groups.

**Fig. 3:**
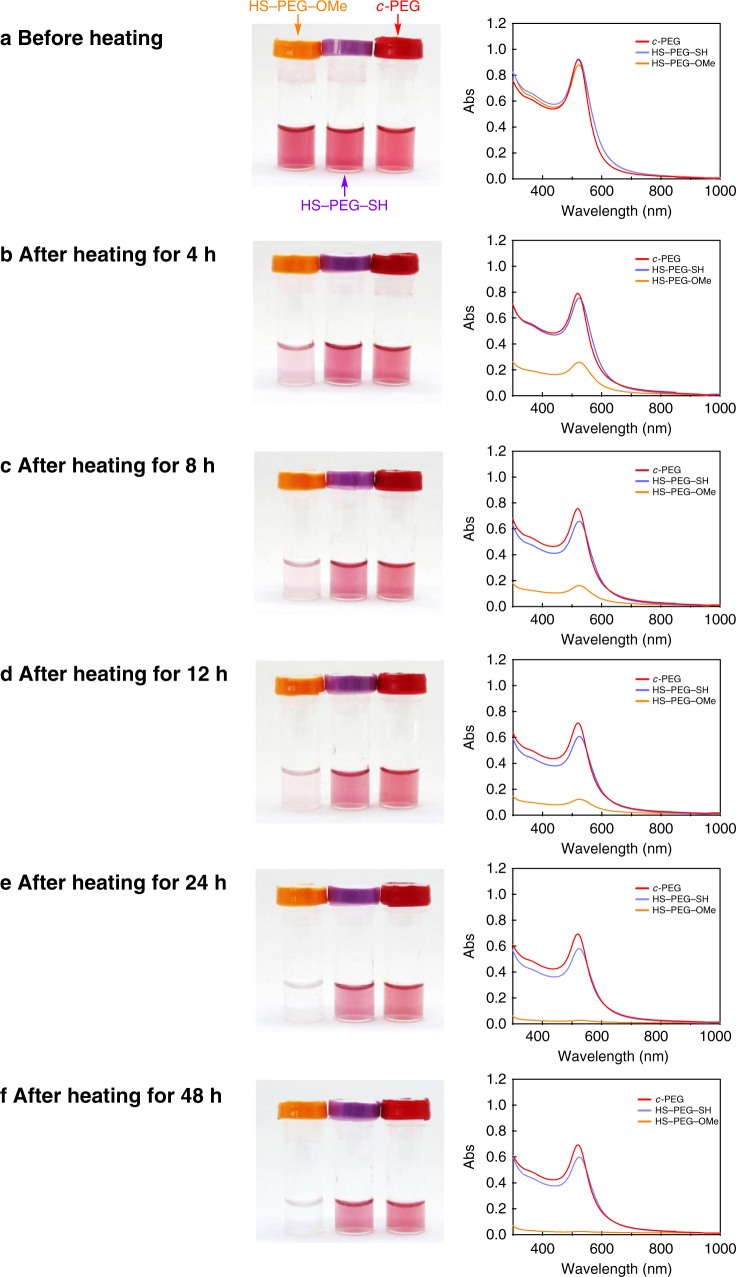
Time course heating test. Photographs and UV–Vis spectra of AuNPs_15_/HS–PEG_3k_–OMe (orange), AuNPs_15_/HS–PEG_2k_–SH (purple), and AuNPs_15_/*c*-PEG_3k_ (red). **a** Before heating and after heating for **b** 4, **c** 8, **d** 12, **e** 24, and **f** 48 h at 85 °C. Rel. Abs was 2% for AuNPs_15_/HS–PEG_3k_–OMe, 64% for AuNPs_15_/HS–PEG_2k_–SH, and 75% for AuNPs_15_/*c*-PEG_3k_ after 48 h.

Furthermore, AuNPs_15_/HS–PEG_2k_–SH was also tested against heating at 85 °C, where the polymers are anchored to the surface at both chain ends (Fig. 3)^31,32^. After 48 h, Rel. Abs was only 2% for AuNPs_15_/HS–PEG_3k_–OMe, 64% for AuNPs_15_/HS–PEG_2k_–SH, and 75% for AuNPs_15_/*c*-PEG_3k_. Both AuNPs_15_/HS–PEG_2k_–SH and AuNPs_15_/*c*-PEG_3k_ showed good long-term stability at high temperature. The high stability of AuNPs_15_/HS–PEG_2k_–SH was likely caused by that even when one chain end of HS–PEG–SH is liberated from the surface of AuNPs by heating, the polymer is held by other chain end, preventing the complete removal from AuNPs. In any case, *c*-PEG_3k_ gave the best result. All of the above experiments suggest that *c*-PEG endows AuNPs with significant dispersion stability against freezing, lyophilization, and heating.

In addition, mixtures of *c*-PEG with MeO–PEG–OMe or HS–PEG–OMe were tested against heating. Thus, MeO–PEG_3k_–OMe (0.225, 0.125, or 0.025 wt%) and *c*-PEG_3k_ (0.025, 0.125, or 0.225 wt%, respectively) were added to AuNPs_30_ to form AuNPs_30_/MeO–PEG_3k_–OMe/*c*-PEG_3k_ with a total PEG concertation of 0.25 wt%. The mixtures were heated at 85 °C for 4 h, resulting in an increase of Rel. Abs with the proportion of *c*-PEG (Rel. Abs = 67, 76, or 81%, respectively) shown in Supplementary Fig. 9. When HS–PEG_3k_–OMe (0.225, 0.125, or 0.025 wt%) and *c*-PEG_3k_ (0.025, 0.125, or 0.225 wt%, respectively) were used, Rel. Abs significantly decreased even for the lowest concentration of HS–PEG_3k_–OMe (Rel. Abs = 17, 17, or 46%, respectively) shown in Supplementary Fig. 10. This indicates that the presence of thiol and/or disulfide at high temperature stimulates the dissolution of AuNPs, and thus HS–PEG–OMe is not a proper capping agent against heating.

**Fig. 4:**
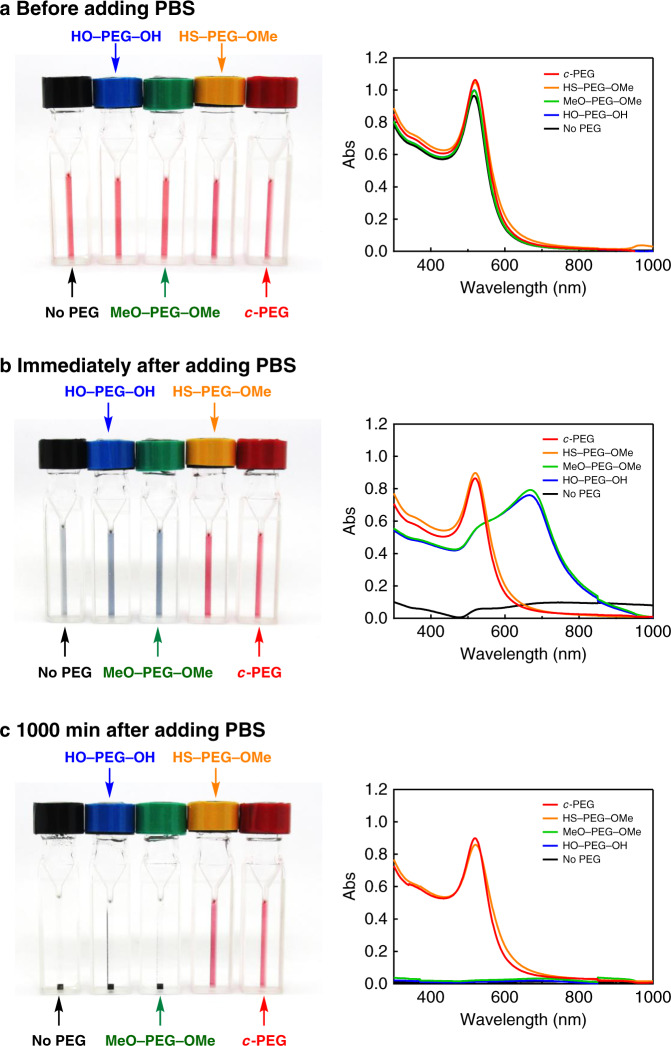
Physiological condition test. Photographs and UV–Vis spectra of AuNPs_15_/No PEG (black), AuNPs_15_/HO–PEG_3k_–OH (blue), AuNPs_15_/MeO–PEG_3k_–OMe (green), AuNPs_15_/HS–PEG_3k_–OMe (orange), and AuNPs_15_/*c*-PEG_3k_ (red). **a** Before, **b** immediately after, and **c** 1000 min after the addition of a tenfold-concentrated PBS solution. The resulting dispersions were pH 7.4 and 150 mM of NaCl.

**Fig. 5:**
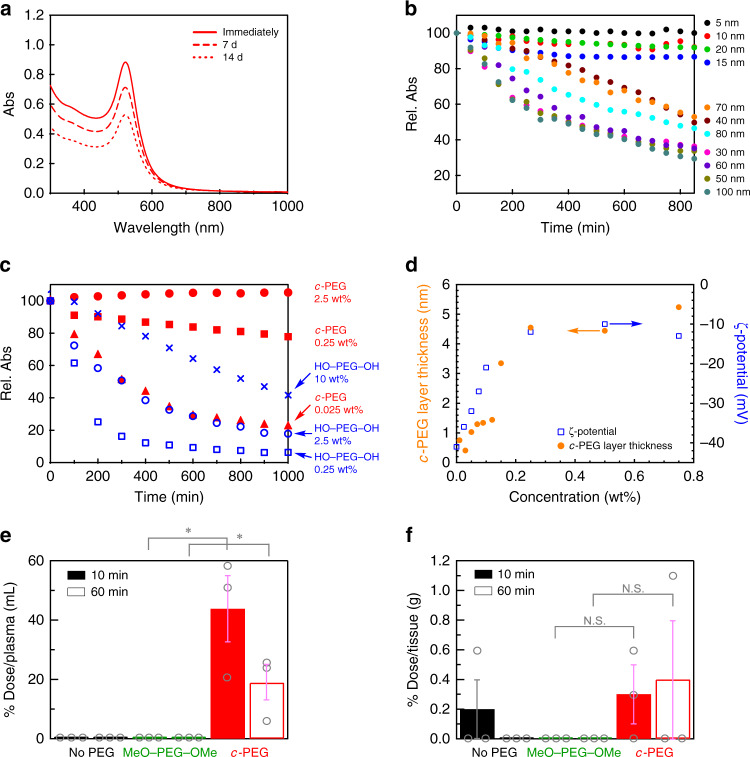
Properties of AuNPs/PEG. **a** UV–Vis spectra of AuNPs_15_/*c*-PEG_3k_ immediately after, 7 d after, and 14 d after the addition of a tenfold-concentrated PBS solution. Rel. Abs was 81% after 7 d and 60% after 14 d. **b** Time course of Rel. Abs of AuNPs/*c*-PEG_3k_ in a physiological condition of pH 7.4 and 150 mM of NaCl. The diameter of AuNPs was 5–100 nm. **c** Time course of Rel. Abs of AuNPs_5_ in the physiological condition in the presence of HO–PEG_5k_–OH or *c*-PEG_5k_ with various concentrations. HO–PEG_5k_–OH, 0.25 wt% (blue square); 2.5 wt% (blue circle); 10 wt% (blue cross). *c*-PEG_5k_, 0.025 wt% (red triangle); 0.25 wt% (red square); 2.5 wt% (red circle). **d** *c*-PEG layer thickness and ζ-potential for AuNPs_15_/*c*-PEG_3k_ with various concentration of *c*-PEG_3k_. Biodistribution of Au in **e** blood and **f** tumor of mice after 10 and 60 min of the injection of AuNPs_5_/No PEG, AuNPs_5_/MeO–PEG_3k_–OMe, and AuNPs_5_/*c*-PEG_3k_. Data represent mean ± s.e. from three independent experiments performed in triplicate. *p < 0.05; N.S. = not significant. Source data for (**e**, **f**) are provided as a Source data file.

Physiological conditions, which contain NaCl and other electrolytes, are also known to cause aggregation due to the increased ionic strength to form a salt with the citrate on the AuNPs surface to partially neutralize the charges^13,33^. Based on the discovered enhanced dispersion stability of AuNPs provided by *c*-PEG, that in a physiological condition was examined for potential biological applications. Thus, the dispersion stability of AuNPs_15_/No PEG, AuNPs_15_/HO–PEG_3k_–OH, AuNPs_15_/MeO–PEG_3k_–OMe, AuNPs_15_/HS–PEG_3k_–OMe, and AuNPs_15_/*c*-PEG_3k_ was tested at a typical physiological condition of pH = 7.4 and 150 mM of NaCl. A tenfold-concentrated phosphate buffer saline (PBS) solution (0.06 mL) was added to the AuNPs_15_/PEG dispersions (0.54 mL) form the intended physiological condition. The color of AuNPs_15_/No PEG, AuNPs_15_/HO–PEG_3k_–OH, and AuNPs_15_/MeO–PEG_3k_–OMe changed to grayish blue immediately after the addition of the concentrated PBS solution and eventually precipitated (Fig. 4). On the contrary, no color change was observed for AuNPs_15_/HS–PEG_3k_–OMe and AuNPs_15_/*c*-PEG_3k_ even after 1000 min. UV–Vis spectra showed that there is no absorption from the SPR of AuNPs_15_/No PEG, AuNPs_15_/HO–PEG_3k_–OH, and AuNPs_15_/MeO–PEG_3k_–OMe due to the precipitation (Rel. Abs ≤ 2% in Table 2), while AuNPs_15_/HS–PEG_3k_–OMe (Rel. Abs = 83%) and AuNPs_15_/*c*-PEG_3k_ (Rel. Abs = 88%) did not exhibited a significant change from those before the addition of the concentrated PBS solution. The slight decrease in Rel. Abs was caused by the dilution of the AuNPs dispersion (0.54 mL) with the concentrated PBS solution (0.06 mL). It is assumed that the surface of AuNPs was protected by the physisorption of *c*-PEG not to contact with other nanoparticles even when AuNPs lose the repulsive surface charges by the increased ionic strength in the physiological condition, leading to the suppression of aggregation. Furthermore, AuNPs_15_/*c*-PEG_3k_ was kept under the physiological condition at 37 °C for an extended period. Rel. Abs was 81% after 7 d and 60% after 14 d, exhibiting a reasonable long-term stability (Fig. 5a and Supplementary Fig. 11).

The size-dependence of AuNPs was then examined by varying from 5 to 100 nm using *c*-PEG_3k_ for the physiological condition test. In the result, AuNPs_20_ and smaller sizes persisted dispersion in the presence of *c*-PEG_3k_ (Fig. 5b and Supplementary Fig. 12). On the other hand, AuNPs_30_ and larger sizes were not sufficiently stabilized, and the color fainted after 850 min. It is known that AuNPs with a smaller size form a more stable dispersion, and the dispersion stability gradually decreases by increasing the size^14^. Thus, *c*-PEG_3k_, which has a diameter of 7.4 nm when the molecule forms an ideal right circular conformation, showed size-dependent stabilization with a threshold between AuNPs_20_ and AuNPs_30_. Additionally, the dependence on the *c*-PEG size was also tested by using *c*-PEG_1k_, *c*-PEG_5k_, and *c*-PEG_10k_ with a diameter of 2.2, 14, and 28 nm, respectively (Table 1). However, no significant difference was observed (Supplementary Fig. 13); all of *c*-PEG_1k_, *c*-PEG_5k_, and *c*-PEG_10k_ gave enhanced dispersion stability to AuNPs_15_. On the contrary, 18-crown-6 ether with molecular weight of 264 Da with 0.7 nm in the estimated diameter did not give dispersion stability, and AuNPs_15_ completely precipitated. Thus, the dispersion stabilization depended on both sizes of AuNPs and *c*-PEG.

Moreover, the PEG concentration-dependence of the dispersion stability was investigated. The *c*-PEG_5k_ concentration was changed from 0.025 to 2.5 wt% for AuNPs_5_ (0.05 mg/mL) in the physiological condition. As shown in Fig. 5c, Rel. Abs decreased to 22% after 1000 min for *c*-PEG_5k_ solutions with 0.025 wt%. However, when the *c*-PEG concertation was 0.25 wt%, the decrease in Rel. Abs was suppressed to 78%. In the case of 2.5 wt% of *c*-PEG_5k_, the absorption essentially quantitatively remained. In this regard, the weight ratio of AuNPs_5_/PEG was 1/500 for 2.5 wt% of the PEG concentration. The concentration-dependence of HO–PEG_5k_–OH was also tested for comparison. In the result, the absorption spectra of AuNPs_5_/HO–PEG_5k_–OH showed a strong declination even with the PEG concentration of 10 wt% (Rel. Abs = 42%).

### DLS and ζ-potential

**Fig. 6:**
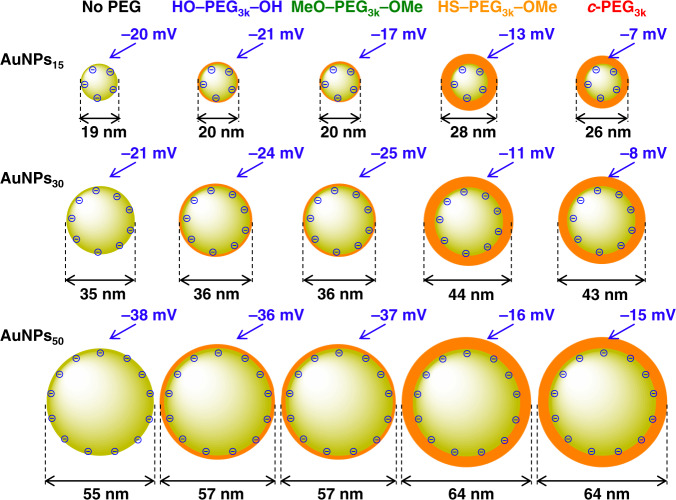
Schematic illustrations of the DLS size and ζ-potential of AuNPs/No PEG, AuNPs/HO–PEG_3k_–OH, AuNPs/MeO–PEG_3k_–OMe, AuNPs/HS–PEG_3k_–OMe, and AuNPs/*c*-PEG_3k_. The size of AuNPs was 15, 30, and 50 nm, and the concentration of PEG was 0.25 wt%. The size ratio of AuNPs and the PEG layer in the figure represents the actual ratio.

In order to investigate the cause of the dispersion stabilization by *c*-PEG, DLS and ζ-potentials were measured using AuNPs_15_, AuNPs_30_, and AuNPs_50_ with no PEG, HO–PEG_3k_–OH, MeO–PEG_3k_–OMe, HS–PEG_3k_–OMe, and *c*-PEG_3k_ at a polymer concentration of 0.25 wt%. The DLS size was nearly identical or slightly increased by 2 nm or less upon the addition of HO–PEG_3k_–OH and MeO–PEG_3k_–OMe compared to corresponding AuNPs/No PEG (Fig. 6). In the case of AuNPs_15_, the diameter was 19 nm for AuNPs_15_/No PEG and 20 nm for AuNPs_15_/HO–PEG_3k_–OH and AuNPs_15_/MeO–PEG_3k_–OMe. In contrast, by the addition of HS–PEG_3k_–OMe or *c*-PEG_3k_, the size was significantly increased by 7 nm or more. For example, the diameter of AuNPs_15_ increased to 28 and 26 nm for AuNPs_15_/HS–PEG_3k_–OMe and AuNPs_15_/*c*-PEG_3k_, respectively. The similar trends in the change in the diameter were observed for AuNPs_30_ and AuNPs_50_, suggesting that HS–PEG_3k_–OMe and *c*-PEG_3k_ have a stronger affinity to the surface of AuNPs and form a thicker layer. Because the surface of AuNPs is coated with citrate, the ζ-potential of AuNPs/No PEG showed negative values such as –20 mV for AuNPs_15_. It was reported that the electrically neutral PEG layer on the surface shields the negative charges of citrate to decrease in the magnitude of the ζ-potential^34^. While the ζ-potential of AuNPs/HO–PEG_3k_–OH and AuNPs/MeO–PEG_3k_–OMe did not significantly change from corresponding AuNPs/No PEG, that of AuNPs/*c*-PEG_3k_ strongly attenuated, which was even better shielding than AuNPs/HS–PEG_3k_–OMe in some cases such as AuNPs_15_/HS–PEG_3k_–OMe (–13 mV) and AuNPs_15_/*c*-PEG_3k_ (–7 mV). Thus, the results of the increased diameter and reduced ζ-potential for AuNPs/*c*-PEG suggest the strong interaction of *c*-PEG to AuNPs and the formation of a PEG layer with a decent density on the basis of the cyclic topology^24^.

Furthermore, the *c*-PEG concentration-dependence of the layer thickness and ζ-potential were determined for AuNPs_15_/*c*-PEG_3k_ (Fig. 5d). Both of the thickness and ζ-potential gradually increased and essentially saturated at around 4.5 nm and –12 mV, respectively, at a PEG concentration of 0.25 wt%, suggesting this concentration is optimal. The grafting density (*σ*) was calculated based on the PEG layer thickness using the following formulae as reported in previous studies (Supplementary Table 2)^35–37^. Thus, the surface concentration (Γ), average distance between grafted PEG chains (*D*), and *σ* were calculated by:

1 $${\Gamma} = \rho t$$

2 $$D = \left( {M/{\Gamma}N_{\mathrm{A}}} \right)^{1/2}$$

3 $$\sigma = \left( {a/D} \right)^2$$

where *a* is the size of a PEG monomer unit (3.5 Å), *ρ* is the density of PEG (1.1 g/mL), *t* is the thickness of the PEG layer by DLS, *N*_A_ is Avogadro’s number, and *M* is the molecular weight of PEG. Despite that the calculation is designated for chemisorption, we consider this helps deducing the state and environment of *c*-PEG at the AuNPs surface. In the result, *σ* was found to be 0.13 chains/nm^2^ at the *c*-PEG concentration of 0.25 wt%. This value may be comparable to 0.20–3.14 chains/nm^2^ reported for HS–PEG–OMe that was determined by a different method, in which the adsorbed PEG molecules was quantitatively removed from AuNPs and analyzed by HPLC^32^. We also attempted this quantitative removal as well as measuring the unadsorbed *c*-PEG in the supernatant^38^. However, these methods need to isolate PEGylated AuNPs from the excess PEG molecules, but this was not possible for *c*-PEG-physiosorbed AuNPs. Thus, repeated centrifugation or ultrafiltration removed *c*-PEG from AuNPs, suggesting that the present physisorption system is dynamic in contrast to the gold–sulfur chemisorption, despite providing the good dispersion stability. In addition, the estimated numbers of PEG molecules and Au atoms present per one AuNP are listed in Supplementary Table 3. For example, in the case of AuNPs_15_ and PEG with *M*_n_ of 3000 Da at a concentration of 0.25 wt%, it is estimated that an average of 10.5 × 10^4^ Au atoms form a nanoparticle^39^, and 38.2 × 10^4^ PEG molecules exist per one nanoparticle.

### FT-IR

In order to determine the interaction between the surface of AuNPs and PEG, FT-IR spectroscopy was performed on AuNPs_15_/No PEG, AuNPs_15_/MeO–PEG_3k_–OMe, and AuNPs_15_/*c*-PEG_3k_. The samples were prepared by moderately removing excess PEG and sodium citrate by ultrafiltration followed by lyophilization. Lyophilization was necessary because water has strong absorptions in the intended wavenumber range. Although FT-IR spectroscopy of the lyophilized samples may not fully reflect the aqueous dispersion state of AuNPs/PEG, the interactions existing between AuNPs and PEG were expected to be investigated. The spectra showed noticeable difference in absorption wavenumbers between AuNPs_15_/MeO–PEG_3k_–OMe and AuNPs_15_/*c*-PEG_3k_ (Supplementary Fig. 14 and Supplementary Table 4). For example, the CH_2_ rocking and twisting of PEG were higher in wavenumber for AuNPs_15_/*c*-PEG_3k_ (961 cm^–1^) than AuNPs_15_/MeO–PEG_3k_–OMe (957 cm^–1^)^40^. Moreover, the C–O and C–C stretching of PEG was also higher for AuNPs_15_/*c*-PEG_3k_ (1100 cm^–1^) than AuNPs_15_/MeO–PEG_3k_–OMe (1094 cm^–1^), where the C–O bonds likely interacted to Na^+^ of sodium citrate. AuNPs_15_/MeO–PEG_3k_–OMe exhibited an extra peak at 1455 cm^–1^ that can also be found in MeO–PEG_3k_–OMe without AuNPs. Thus, this peak likely arose from the effect of the chain end. The asymmetric stretching of COO^–^ of sodium citrate appeared at 1575 cm^–1^ for AuNPs_15_/No PEG, 1578 cm^–1^ for AuNPs_15_/MeO–PEG_3k_–OMe, and 1581 cm^–1^ for AuNPs_15_/*c*-PEG_3k_. This suggests that MeO–PEG_3k_–OMe interacts with Na^+^ to cause the change in the COO^–^ stretching wavenumber, and moreover, *c*-PEG_3k_ results in a stronger interaction. In addition, the peak at 1648–1649 cm^–1^, not much different in all the samples, was from the asymmetric stretching of COO^–^ of Au-coordinated citrate^41^.

### Animal experiments

Applications of nano-sized particles have been extensively developed for bio-medical area, and, especially, AuNPs are widely utilized into biosensing, diagnosis, and in vivo drug delivery^42–46^. Thus, further research into the plasma clearance and tumor accumulation by using tumor bearing mouse would clarify the potential usability of AuNPs/*c*-PEG in vivo. As seen in Fig. 5e. AuNPs_5_/*c*-PEG_3k_ exhibited slow blood clearance (43.8% dose/plasma at 10 min, and 18.8% dose/plasma at 60 min), while no stable circulations were observed for AuNPs_5_/No PEG and AuNPs_5_/MeO–PEG_3k_–OMe, indicating the topology-dependent utility of surface-modification through *c*-PEG. Tumor accumulation also represented the advantage of AuNPs_5_/*c*-PEG_3k_ (Fig. 5f). However, on the basis of the previous reports^47–49^, the absolute amount of AuNPs_5_/*c*-PEG_3k_ accumulated into tumor was relatively low values. In fact, AuNPs_5_/*c*-PEG_3k_ in this study is assumed to be accumulated by the enhanced permeability and retention (EPR) effect^50,51^, and the relationship between particle size and time course are fundamentally important to obtain EPR-driven tumor accumulation, especially vs hypervascular tumors, i.e., murine colon adenocarcinoma (C26)^47,52^. Therefore, the increments in tumor accumulation of nano-sized particle through EPR effect might require additional fine tuning of AuNPs, e.g., particle size, and such additional modification is ongoing in our group. Overall, these results highlight the importance and usability of *c*-PEG as a stabilizer for metal nanoparticle, and our in vivo experiments exhibit the potential effectiveness of AuNPs/*c*-PEG, prepared through simple physisorption of *c*-PEG against AuNPs, for bio-medical applications.

## Discussion

Concerning the cause of the strong affinity of *c*-PEG to the surface of AuNPs, the less entropic penalty upon adsorption is expected to be the major factor. In other words, the number of conformations that unadsorbed *c*-PEG can take is less than that of the unadsorbed linear PEG (HO−PEG−OH and MeO−PEG−OMe) due to their topology. Thus, the entropic penalty upon adsorption onto the surface of AuNPs is smaller for *c*-PEG, where the adsorption is an entropically unfavorable and enthalpically favorable process. Since HO−PEG−OH, MeO−PEG−OMe, and *c*-PEG are consisted of the same PEG repeating units, their enthalpic energy released upon adsorption should be essentially identical, leading to that the adsorption of *c*-PEG is more favorable than HO−PEG−OH and MeO−PEG−OMe in terms of the free energy to form the thicker PEG layer for the enhanced dispersion stabilization.

The stronger affinity of cyclic polymers was also observed against silica nanoparticles^38,53^. Moreover, the stronger adsorption of cyclic polymers onto a surface have been suggested by theoretical and computational studies^54–57^. Cosgrove and coworkers conducted a detailed study on the difference between the surface adsorption of cyclic and linear polymers from solutions by the extended lattice theory (Scheutjens−Fleer (SF) theory)^58^. When the surface adsorption energy parameter (*χ*_s_) is small, a larger amount of the cyclic polymers is adsorbed than the linear analogs. The difference in the adsorption efficiency between linear and cyclic polymers becomes small with increasing *χ*_s_. This result indicates that the difference between the linear and cyclic chains is negligible when the adsorption is strongly controlled by enthalpy, while the cyclic chain is advantageous when the adsorption enthalpy and entropy are comparable.

In summary, we showed that the drastic enhancement in the dispersion stability of AuNPs by physisorption of *c*-PEG. This methodology allows for AuNPs dispersions to be frozen, lyophilized, heated, and exposed to physiological conditions with even better stability than the most commonly used chemisorption of HS–PEG–OMe. Moreover, the AuNPs/*c*-PEG system was applied for an animal experiment, finding the advantages of AuNPs_5_/*c*-PEG_3k_ in blood circulation in contrast to AuNPs_5_/No PEG and AuNPs_5_/MeO–PEG_3k_–OMe. The combination of the dispersion stability against high temperature and physiological conditions, coupled with the accumulation in tumor, would be most suitable to future applications toward photothermal therapy using gold nanoparticles and nanorods. Furthermore, cyclization and physisorption require no chemical modification on the repeating units of the polymer chain, which is excellent match for the biocompatibility of PEG. Likewise, the physisorption would allow for expedient postfunctionalization through chemisorption.

## Methods

### Materials

Gold nanoparticles (AuNPs) with a diameter of 5, 10, 15, 20, 30, 40, 50, 60, 70, 80, and 100 nm (0.05 mg/mL in aqueous 2 mM sodium citrate, nanoComposix), 4-toluenesulfonyl chloride (TsCl) (>98.0%, Junsei Chemical Co., Ltd.), sodium chloride (NaCl) (>99.0%, Kanto Chemical Co., Inc.), potassium hydroxide (KOH) (>85.5%, Kanto Chemical Co., Inc.), iodomethane (>99.5%, Kanto Chemical Co., Inc.), magnesium sulfate (MgSO_4_) (>98.0%, Kanto Chemical Co., Inc.), *n*-heptane (>99.0%, Kanto Chemical Co., Inc.), chlorobenzene (>99.0%, Nacalai Tesque, Inc.), 18-crown 6-ether (Tokyo Chemical Industry Co., Ltd.), and dithiolated poly(ethylene glycol) (HS–PEG_2k_–SH) (Funakoshi Co., Ltd.) were used as received. Tetrahydrofuran (THF), dehydrated stabilizer free (>99.5%, Kanto Chemical Co., Inc.), dichloromethane (CH_2_Cl_2_) (>99.0%, Kanto Chemical Co., Inc.), and chloroform (CHCl_3_) (>99.0%, Kanto Chemical Co., Inc.) were purified by a solvent purification system (MBRAUN MB-SPS-Compact). Poly(ethylene glycol) 950–1050 (HO–PEG_1k_–OH) (Sigma-Aldrich), poly(ethylene glycol) 2,000 (HO–PEG_3k_–OH) (Sigma-Aldrich), poly(ethylene glycol) 4,000 (HO–PEG_5k_–OH) (Kanto Chemical Co., Inc.), poly(ethylene glycol) 6,000 (HO–PEG_10k_–OH) (Kanto Chemical Co., Inc.) were purified by passing through a silica gel column using CHCl_3_/MeOH (90/10, v/v) as an eluent. Thiolated poly(ethylene glycol) (HS–PEG_3k_–OMe) (Funakoshi Co., Ltd.) was purified by recycling preparative SEC. The molecular weights of PEG in the catalogs deviated from our measurement, and the measurement values are used in this paper.

### NMR

^1^H (400 MHz) and ^13^C NMR (100 MHz) were measured in CDCl_3_ or D_2_O using a JEOL JNM-ESC400 instrument at ambient temperature.

### SEC

Size exclusion chromatography (SEC) was performed at 40 °C with THF (flow rate, 1.0 mL/min) using a Shodex GPC-101 gel permeation chromatography system (Shodex DU-2130 dual pump, Shodex RI-71 reflective index detector, and Shodex ERC-3125SN degasser) equipped with a Shodex KF-G guard column (4.6 mm × 10 mm; pore size, 8 μm) and two Shodex KF-804L columns (8 mm × 300 mm) in series. Polystyrene standard samples were used for calibration.

### Recycling preparative SEC

A Japan Analytical Industry LC-908 recycling preparative HPLC system (Hitachi L-7110 pump and JAI RI detector RI-5) was used. JAIGEL-2H and 3H columns and a pre-column were connected in series. CHCl_3_ was used as the solvent, and the flow rate was set at 3.5 mL/min.

### MALDI-TOF MS

Matrix-assisted laser desorption/ionization time-of-flight mass spectrometry (MALDI-TOF MS) was carried out using an ABSCIEX TOF/TOF 5800 mass spectrometer at the Open Facility, Hokkaido University. A PEG sample (1.5 mg/mL, 10 µL) was mixed with a matrix (anthralin, 40 mg/mL, 25 µL) and ionization regent (silver trifluroroacetate, 40 mg/mL, 10 µL), and 0.4 µL of the mixture was drop cast on an Opti-TOF 384-Well Insert (123 × 81 mm) plate.

### UV–Vis Spectroscopy

Absorption spectra were measured on a JASCO Ubset V-670 spectrophotometer at 25 °C in deionized water using a micro quartz cell (M25-UV-2, GL Science Inc. Japan).

### Synthesis of *c*-PEG

HO–PEG_1k_–OH, HO–PEG_3k_–OH, HO–PEG_5k_–OH, and HO–PEG_10k_–OH were cyclized according to a previous report by Cooke et al.^26^. Typically, HO–PEG_3k_–OH (5.0 g, 2.5 mmol) and TsCl (0.63 g, 3.3 mmol) were dissolved in a dry THF (100 mL) by using a 300 mL three-neck flask and added dropwise to a stirring suspension of KOH (3.3 g) in THF/*n*-heptane (75/25, 100 mL) through a syringe pump at 40 °C under Ar. The addition was conducted over 144 h at a rate of 0.7 mL/h. The reaction mixture was stirred for another 24 h at 40 °C. The resulting suspension was filtered and concentrated under reduced pressure. The residue was redissolved in CH_2_Cl_2_ and washed with a saturated NaCl solution three times. The combined organic phase was dried over MgSO_4_ and concentrated under reduced pressure, and the residue was passed through a silica gel column using CHCl_3_/MeOH (90/10 v/v) as an eluent. The obtained solid was dissolved in CH_2_Cl_2_, and *n*-heptane was added to the solution until becoming cloudy. The mixture was heated to 40 °C and cooled to 25 °C gave two separated phases. The *n*-heptane phase was collected and concentrated under reduced pressure. This step was conducted three more times to isolate *c*-PEG_3k_ (420 mg, 8.4%) as colorless solid.

The expected diameter of *c*-PEG, when the polymer forms an ideal right circular conformation, as well as that of 18-crown-6 ether, was determined by the simple geometric calculation (circumference = π × diameter) based on the length of HO–PEG–OH.

### Synthesis of MeO–PEG–OMe

Dimethylation of HO–PEG–OH was performed using a previously reported procedure^26^. For example, a suspension of KOH (6.9 g) in chlorobenzene (60 mL) in a 300 mL three-neck flask was added dropwise to a solution of HO–PEG_3k_–OH (5.00 g) in chlorobenzene (50 mL), followed by the slow addition of iodomethane (1.05 g) over 50 min under N_2_. After the addition was completed, the mixture was stirred at 25 °C for 24 h. The resulting suspension was diluted with CH_2_Cl_2_ and filtered, and the filtrate was concentrated under reduced pressure. The residue was dissolved in CH_2_Cl_2_ and washed with a saturated NaCl solution three times, dried over MgSO_4_, and concentrated under reduced pressure. The residue was dissolved in CHCl_3_, passed through a silica gel column using CHCl_3_/MeOH (90/10 v/v) as an eluent, and concentrated under reduced pressure to give MeO–PEG_3k_–OMe (3.26 g, 65%) as white solid.

### Preparation of AuNPs/HO–PEG–OH, AuNPs/MeO–PEG–OMe, AuNPs/HS–PEG–OMe, AuNPs/HS–PEG–SH, and AuNPs/*c*-PEG

Typically, HO–PEG_3k_–OH (25 mg) was added to an AuNPs_15_ dispersion (1.0 mL) in a 2.0 mL vial, and PEG was dissolved by vortex mixing for 1 min to form AuNPs_15_/HO–PEG_3k_–OH with a PEG concentration of 2.5 wt%. The concentration of PEG was controlled by changing the amount of PEG.

### Dispersion stability against freezing

AuNPs_15_/No PEG, AuNPs_15_/HO–PEG_3k_–OH, AuNPs_15_/MeO–PEG_3k_–OMe, AuNPs_15_/HS–PEG_3k_–OMe, and AuNPs_15_/*c*-PEG_3k_ (1.0 mL) with a PEG concentration of 2.5 wt% were frozen in a household refrigerator overnight. The frozen samples were melted at ambient temperature.

### Dispersion stability against lyophilization

AuNPs_15_/No PEG, AuNPs_15_/HO–PEG_3k_–OH, AuNPs_15_/MeO–PEG_3k_–OMe, AuNPs_15_/HS–PEG_3k_–OMe, and AuNPs_15_/*c*-PEG_3k_ (1.0 mL) with a PEG concentration of 2.5 wt% were frozen in liquid nitrogen for 1 min and lyophilized overnight under reduced pressure. Deionized water (1.0 mL) was added to the residues for redispersion.

### Dispersion stability against heating

AuNPs_15_/No PEG, AuNPs_15_/HO–PEG_3k_–OH, AuNPs_15_/MeO–PEG_3k_–OMe, AuNPs_15_/HS–PEG_3k_–OMe, and AuNPs_15_/*c*-PEG_3k_ (1.0 mL) with a PEG concentration of 0.25 wt% were placed in a 15 mL vial with a lid to enclose air and prevent evaporation of water and heated for 4 h in a water bath set at 85 °C. Likewise, AuNPs_15_/HS–PEG_3k_–OMe, AuNPs_15_/HS–PEG_2k_–SH, and AuNPs_15_/*c*-PEG_3k_ (1.0 mL) with a PEG concentration of 0.25 wt% were placed in a 15 mL vial with a lid and heated for 48 h in a water bath set at 85 °C. Similarly, the heating test of AuNPs_30_/MeO–PEG_3k_–OMe/*c*-PEG_3k_ and AuNPs_30_/HS–PEG_3k_–OMe/*c*-PEG_3k_ was performed. Typically, MeO–PEG_3k_–OMe (2.25 mg, 0.225 wt%) and *c*-PEG_3k_ (0.25 mg, 0.025 wt%) were dissolved in AuNPs_30_ dispersion (1.0 mL). The total PEG concentration was 0.25 wt%. The resulting dispersion was placed in a 15 mL vial with a lid and heated for 4 h in a water bath set at 85 °C. Photographs and UV–Vis spectra were recorded at the designated times.

### Dispersion stability against a PBS buffer solution

A tenfold-concentrated phosphate-buffered saline (PBS) solution (pH 7.4, NaCl 1500 mM, Na_2_HPO_4_ 81 mM, NaH_2_PO_4_ 14.7 mM) was prepared in advance. Typically, AuNPs_15_/No PEG, AuNPs_15_/HO–PEG_3k_–OH, AuNPs_15_/MeO–PEG_3k_–OMe, AuNPs_15_/HS–PEG_3k_–OMe, or AuNPs_15_/*c*-PEG_3k_ (0.54 mL) was placed in a micro quartz cuvette to measure an absorption spectrum. Subsequently, the tenfold-concentrated PBS solution (0.06 mL) was added to the cuvette, and the resulting mixture was 0.6 mL with pH 7.4 and 150 mM of NaCl with a PEG concentration of 0.25 wt%. A time-course UV–Vis measurement was performed for 1000 min. For the long-term stability test, AuNPs_15_/*c*-PEG_3k_ was kept at 37 °C for 14 d after the addition of the tenfold-concentrated PBS solution.

### Stabilization of AuNPs with various diameters

*c*-PEG_3k_ (1.5 mg) was added to an AuNPs dispersion (0.54 mL) with a diameter of 5, 10, 15, 20, 30, 40, 50, 60, 70, 80, or 100 nm in a 2 mL vial and dissolved by vortex mixing for 1 min. Subsequently, the tenfold-concentrated PBS solution (0.06 mL) was added to AuNPs/*c*-PEG, and the resulting mixture was 0.6 mL with pH 7.4 and 150 mM of NaCl with a PEG concentration of 0.25 wt%. A time-course UV–Vis measurement was performed for 850 min.

### PEG concentration-dependent stabilization of AuNPs

The tenfold-concentrated PBS solution (0.06 mL) was added to AuNPs_5_/HO–PEG_5k_–OH (0.54 mL) or AuNPs_5_/*c*-PEG_5k_ (0.54 mL), and the resulting mixture was 0.6 mL with pH 7.4 and 150 mM of NaCl with a PEG concentration of 0.25, 2.5, or 10 wt% for AuNPs_5_/HO–PEG_5k_–OH or 0.025, 0.25, or 2.5 wt% for AuNPs_5_/*c*-PEG_5k_. A time-course UV–Vis measurement was performed for 1000 min.

### DLS and ζ-potential

Zetasizer Nano ZS (Malvern Panalytical Ltd.) with a high precision micro quartz cuvette (ZEN2112, Hellma Analytics) and Zetasizer nano cells (DTS1060, Malvern Instruments Ltd.) were used to measure the diameter and ζ-potential, respectively. All measurements were performed at 25 °C with 20 scans at most with a 120 s equilibration time.

### FT-IR

2.0 mL of AuNPs_15_/No PEG (0.25 wt%), AuNPs_15_/MeO–PEG_3k_–OMe (0.25 wt%), or AuNPs_15_/*c-*PEG_3k_ (0.25 wt%) was ultrafiltrated at 4,700 g for 20 min using an Amicon Ultra – 2 mL centrifugal filter Ultracel – 30 K twice. Deionized water was added to a total volume of 2.0 mL, and the dispersion was frozen in liquid nitrogen for 1 min and lyophilized overnight under reduced pressure. The FT-IR spectrum of the lyophilized sample was measured using a PerkinElmer Frontier MIR spectrometer equipped with a single reflection diamond universal attenuated total reflection (ATR) accessory.

### Cell lines and animals

C26 cells (supplied by the National Cancer Center, Tokyo, Japan) were cultured with Dulbecco’s Modified Eagle’s Medium (high glucose, Sigma-Aldrich, D6429) plus 10% fetal bovine serum (FBS, Dainippon Sumitomo Pharma Co., Ltd.). BALB/c female mice, 6-weeks (18–21 g) were obtained from Oriental Yeast Co., Ltd. (Tokyo, Japan) and were allowed to acclimatize for 5 days before inoculation of tumors. Animals were kept in a temperature-controlled room on 12 h/12 h light / dark schedule at 22 °C with food and water ad libitum. A suspension of C26 cells (1.0 × 10^6^ cells/100 μL in PBS) was subcutaneously implanted into BALB/c female mice. Tumor growth was measured by calipebio Ar measurement and tumor volumes were calculated by the following equation: *V* = (*a* × *b*^2^)/2, where *a* refers to the tumor width (mm) and *b* refers to the tumor length (mm). Fourteen-days post-inoculation, the size tumors were reached to 50 mm^3^, animals were separated into *n* = 3 per group and for treatment with: AuNPs_5_/No PEG, AuNPs_5_/MeO–PEG_3k_–OMe, and AuNPs_5_/*c*-PEG_3k_ (dose = 5 mg/kg on a gold basis). The mice were sacrificed 10 and 60 min after i.v, injection. The tumors were excised, washed with PBS and weighed after removing excess fluid. Blood was collected from the inferior vena cava, heparinized, and centrifuged to obtain the plasma. Acid digestion of all samples was carried out using ca. 5.0 mL of 30% HNO_3_/35% HCl mixture. Obtained samples were dissolved in 3 vol% HNO_3_ aq. (10 mL) and were filtered by PTFE membrane. Gold concentration was measured by inductivity coupled plasma optical emission spectrometer with yttrium as internal standard. All animal experiments were performed in accordance with the Guidelines for the Care and Use of Laboratory Animals as stated by The University of Tokyo, Tokyo Institute of Technology, and Hokkaido University.

### Reporting summary

Further information on research design is available in the Nature Research Reporting Summary linked to this article.

## Supplementary information

Supplementary Information

Reporting Summary

## Data Availability

The data that support the findings of this study are available from the corresponding author upon reasonable request. Source data are provided with this paper.

## Source data

Source Data

## References

[1] Kelly KL, Coronado E, Zhao LL, Schatz GC (2003). The optical properties of metal nanoparticles: the influence of size, shape, and dielectric environment. J. Phys. Chem. B.

[2] Kim YJ, Johnson RC, Hupp JT (2001). Gold nanoparticle-based sensing of “spectroscopically silent” heavy metal ions. Nano Lett..

[3] Anker JN (2008). Biosensing with plasmonic nanosensors. Nat. Mater..

[4] Hasobe T (2005). Photovoltaic cells using composite nanoclusters of porphyrins and fullerenes with gold nanoparticles. J. Am. Chem. Soc..

[5] Yan N, Xiao C, Kou Y (2010). Transition metal nanoparticle catalysis in green solvents. Coord. Chem. Rev..

[6] Wiley B, Sun YG, Xia YN (2007). Synthesis of silver nanostructures with controlled shapes and properties. Acc. Chem. Res..

[7] Radziuk D, Skirtach A, Sukhorukov G, Shchukin D, Möhwald H (2007). Stabilization of silver nanoparticles by polyelectrolytes and poly(ethylene glycol). Macromol. Rapid Commun..

[8] Dong HC (2008). One-pot synthesis of robust core/shell gold nanoparticles. J. Am. Chem. Soc..

[9] Zhang X, Servos MR, Liu JW (2012). Ultrahigh nanoparticle stability against salt, pH, and solvent with retained surface accessibility via depletion stabilization. J. Am. Chem. Soc..

[10] Kralik M (2014). Adsorption, chemisorption, and catalysis. Chem. Pap..

[11] Zhang G (2009). Influence of anchoring ligands and particle size on the colloidal stability and in vivo biodistribution of polyethylene glycol-coated gold nanoparticles in tumor-xenografted mice. Biomaterials.

[12] Nam S, Parikh DV, Condon BD, Zhao Q, Yoshioka-Tarver M (2011). Importance of poly(ethylene glycol) conformation for the synthesis of silver nanoparticles in aqueous solution. J. Nanopart. Res..

[13] Boisselier E, Astruc D (2009). Gold nanoparticles in nanomedicine: preparations, imaging, diagnostics, therapies and toxicity. Chem. Soc. Rev..

[14] Ling K, Jiang H, Zhang Q (2013). A colorimetric method for the molecular weight determination of polyethylene glycol using gold nanoparticles. Nanoscale Res. Lett..

[15] Seo E (2017). Highly stable Au nanoparticles with double hydrophilic block copolymer templates: correlation between structure and stability. Polym. Chem..

[16] Jia Z, Monteiro MJ (2012). Cyclic polymers: methods and strategies. J. Polym. Sci., Part A: Polym. Chem..

[17] Laurent BA, Grayson SM (2009). Synthetic approaches for the preparation of cyclic polymers. Chem. Soc. Rev..

[18] Kricheldorf HR, Schwarz G (2003). Cyclic polymers by kinetically controlled step-growth polymerization. Macromol. Rapid Commun..

[19] Yamamoto T, Tezuka Y (2015). Cyclic polymers revealing topology effects upon self-assemblies, dynamics and responses. Soft Matter.

[20] Honda S, Yamamoto T, Tezuka Y (2010). Topology-directed control on thermal stability: micelles formed from linear and cyclized amphiphilic block copolymers. J. Am. Chem. Soc..

[21] Honda S, Yamamoto T, Tezuka Y (2013). Tuneable enhancement of the salt and thermal stability of polymeric micelles by cyclized amphiphiles. Nat. Commun..

[22] Baba E, Yatsunami T, Tezuka Y, Yamamoto T (2016). Formation and properties of vesicles from cyclic amphiphilic PS–PEO block copolymers. Langmuir.

[23] Morgese G (2016). Topological polymer chemistry enters surface science: linear versus cyclic polymer brushes. *Angew. Chem*. Int. Ed..

[24] Morgese G (2017). Next-generation polymer shells for inorganic nanoparticles are highly compact, ultra-dense, and long-lasting cyclic brushes. *Angew. Chem*. Int. Ed..

[25] Morgese G, Cavalli E, Rosenboom J-G, Zenobi-Wong M, Benetti EM (2018). Cyclic polymer grafts that lubricate and protect damaged cartilage. *Angew. Chem*. Int. Ed..

[26] Cooke J (1998). Large cyclic poly(oxyethylene)s: chain folding in the crystalline state studied by Raman spectroscopy, X-ray scattering, and differential scanning calorimetry. Macromolecules.

[27] Tang Q, Wu Y, Sun P, Chen Y, Zhang K (2014). Powerful ring-closure method for preparing varied cyclic polymers. Macromolecules.

[28] Liu R (2013). Colorimetric sensing of copper(II) based on catalytic etching of gold nanoparticles. Talanta.

[29] Smith AM (2015). Quantitative analysis of thiolated ligand exchange on gold nanoparticles monitored by ^1^H NMR spectroscopy. Anal. Chem..

[30] Bain CD, Biebuyck HA, Whitesides GM (1989). Comparison of self-assembled monolayers on gold: coadsorption of thiols and disulfides. Langmuir.

[31] Kang T (2016). Mussel-inspired anchoring of polymer loops that provide superior surface lubrication and antifouling aroperties. ACS Nano.

[32] Du Y, Jin J, Liang H, Jiang W (2019). Structural and physicochemical properties and biocompatibility of linear and looped polymer-capped gold nanoparticles. Langmuir.

[33] Pamies R (2014). Aggregation behaviour of gold nanoparticles in saline aqueous media. J. Nanopart. Res..

[34] Rahme K (2013). PEGylated gold nanoparticles: polymer quantification as a function of PEG lengths and nanoparticle dimensions. RSC Adv..

[35] Tao SL, Popat KC, Norman JJ, Desai TA (2008). Surface modification of SU-8 for enhanced biofunctionality and nonfouling properties. Langmuir.

[36] Damodaran VB, Fee CJ, Ruckh T, Popat KC (2010). Conformational studies of covalently grafted poly(ethylene glycol) on modified solid matrices using X-ray photoelectron spectroscopy. Langmuir.

[37] Uz M, Bulmus V, Altinkaya SA (2016). Effect of PEG grafting density and hydrodynamic volume on gold nanoparticle-cell interactions: an investigation on cell cycle, apoptosis, and DNA damage. Langmuir.

[38] Wang Y, Qin W, Qiu D (2014). Small-angle neutron scattering study of cyclic poly(ethylene glycol) adsorption on colloidal particles. Langmuir.

[39] Oh E (2011). Cellular uptake and fate of PEGylated gold nanoparticles is dependent on both cell-penetration peptides and particle size. ACS Nano.

[40] Vrandečić NS, Erceg M, Jakić M, Klarić I (2010). Kinetic analysis of thermal degradation of poly(ethylene glycol) and poly(ethylene oxide)s of different molecular weight. Thermochim. Acta.

[41] Wulandari P (2015). Characterization of citrates on gold and silver nanoparticles. J. Colloid Interface Sci..

[42] Elsabahy M, Wooley KL (2012). Design of polymeric nanoparticles for biomedical delivery applications. Chem. Soc. Rev..

[43] Sutton D, Nasongkla N, Blanco E, Gao JM (2007). Functionalized micellar systems for cancer targeted drug delivery. Pharm. Res..

[44] Agasti SS (2010). Nanoparticles for detection and diagnosis. Adv. Drug Deliv. Rev..

[45] Knop K, Hoogenboom R, Fischer D, Schubert US (2010). Poly(ethylene glycol) in drug delivery: pros and cons as well as potential alternatives. Angew. Chem., Int. Ed..

[46] Otsuka H, Nagasaki Y, Kataoka K (2003). PEGylated nanoparticles for biological and pharmaceutical applications. Adv. Drug Deliv. Rev..

[47] Cabral H (2011). Accumulation of sub-100 nm polymeric micelles in poorly permeable tumours depends on size. Nat. Nanotechnol..

[48] Bae Y (2005). Preparation and biological characterization of polymeric micelle drug carriers with intracellular pH-triggered drug release property: Tumor permeability, controlled subcellular drug distribution, and enhanced in vivo antitumor efficacy. Bioconjugate Chem..

[49] Cabral H, Nishiyama N, Okazaki S, Koyama H, Kataoka K (2005). Preparation and biological properties of dichloro(1,2-diaminocyclohexane)platinum(II) (DACHPt)-loaded polymeric micelles. J. Control. Release.

[50] Matsumura Y, Maeda H (1986). A new concept for macromolecular therapeutics in cancer chemotherapy: mechanism of tumoritropic accumulation of proteins and the antitumor agent smancs. Cancer Res.

[51] Maeda H, Tsukigawa K, Fang J (2016). A retrospective 30 years after discovery of the enhanced permeability and retention effect of solid tumors: next-generation chemotherapeutics and photodynamic therapy—problems, solutions, and prospects. Microcirculation.

[52] Miura Y (2015). Polymeric micelle platform for multimodal tomographic imaging to detect scirrhous gastric cancer. ACS Biomater. Sci. Eng..

[53] Patel A, Cosgrove T, Semlyen JA (1991). Studies of cyclic and linear poly(dimethylsiloxanes): 30. adsorption studies on silica in solution. Polymer.

[54] Stratouras GK, Kosmas MK (1991). On the density profile of ring chains interacting with a surface. Macromolecules.

[55] Stratouras G, Kosmas M (1992). Are ring polymers adsorbed on a surface more than linear polymers?. Macromolecules.

[56] Zhang L, Xia A, Xu Y (2000). Statics and dynamics of adsorbed ring polymer chains. Eur. Polym. J..

[57] Sikorski A (2001). Computer simulation of adsorbed polymer chains with a different molecular architecture. Macromol. Theory Simul..

[58] van Lent B, Scheutjens J, Cosgrove T (1987). Self-consistent field-theory for the adsorption of ring polymers from solution. Macromolecules.

