# Supplementary material for: Enhanced dispersion stability of gold nanoparticles by the physisorption of cyclic poly(ethylene glycol)
Source: Nat Commun. 2020 Nov 30;11:6089. doi: 10.1038/s41467-020-19947-8 (PMC7705015; doi:10.1038/s41467-020-19947-8)
Supplement: Supplementary file 1 — Supplementary Information [file 41467_2020_19947_MOESM1_ESM.pdf]

# Supplementary information

# Enhanced dispersion stability of gold nanoparticles by the physisorption of cyclic poly(ethylene glycol)

Yubo Wang[1], Jose Enrico Q. Quinsaat[2], Tomoko Ono[2], Masatoshi Maeki[2], Manabu Tokeshi[2], Takuya Isono[2], Kenji Tajima[2], Toshifumi Satoh[2], Shin-ichiro Sato[2], Yutaka Miura[3,4], and Takuya Yamamoto[2*]

[1]Graduate School of Chemical Sciences and Engineering, Hokkaido University, Sapporo, Hokkaido 060–8628, Japan.

[2]Division of Applied Chemistry, Faculty of Engineering, Hokkaido University, Sapporo, Hokkaido 060–8628, Japan.

[3]Department of Bioengineering, Graduate School of Engineering, The University of Tokyo, 7–3–1 Hongo, Bunkyo-ku, Tokyo 113–8656, Japan.

[4]Laboratory for Chemistry and Life Science, Institute of Innovative Research, Tokyo Institute of Technology, 4259 Nagatsutacho, Midori-ku, Yokohama, Kanagawa, 226–8503, Japan.

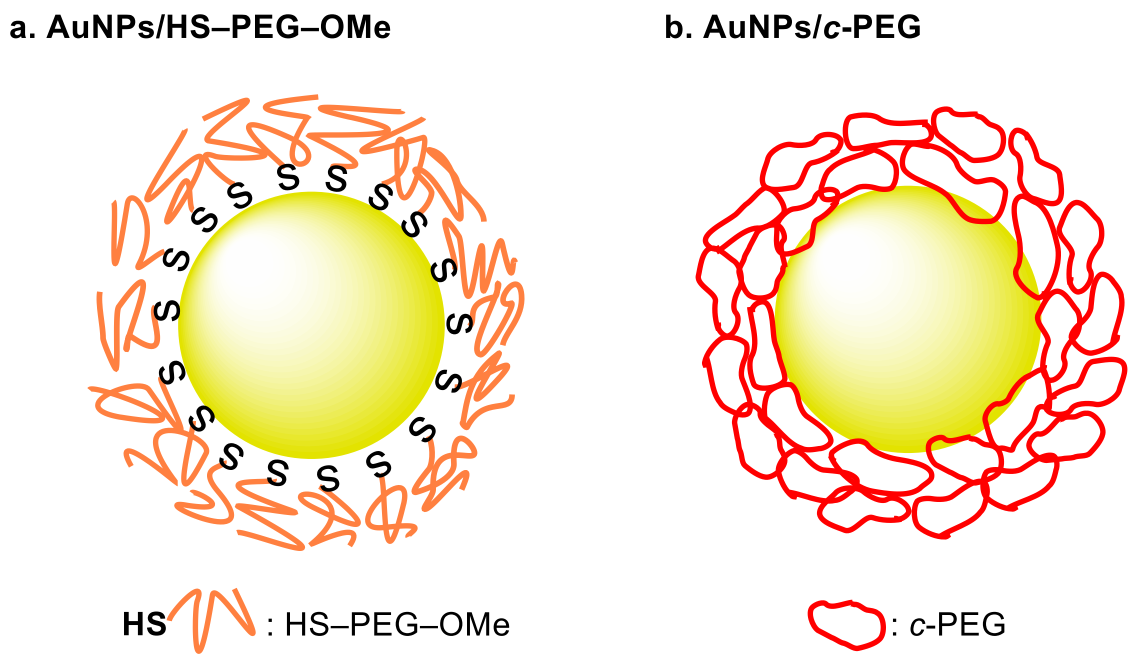


**Supplementary Fig. 1 Schematic illustrations of PEGylated AuNPs. a** AuNPs/HS–PEG–OMe and **b** AuNPs/*c*-PEG.

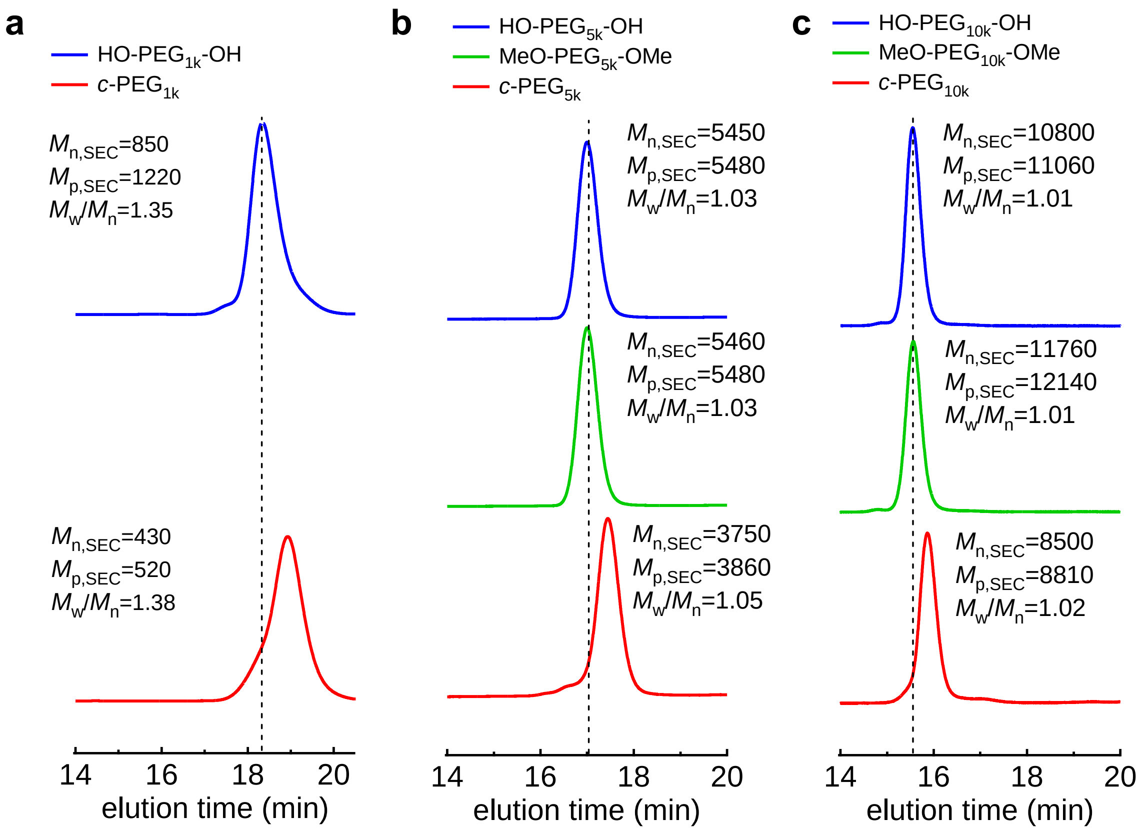


**Supplementary Fig. 2 SEC traces of HO–PEG–OH (blue), MeO–PEG–OMe (green), and *c*-PEG (red).** The molecular weight was **a** 1, **b** 5, and **c** 10 kDa.

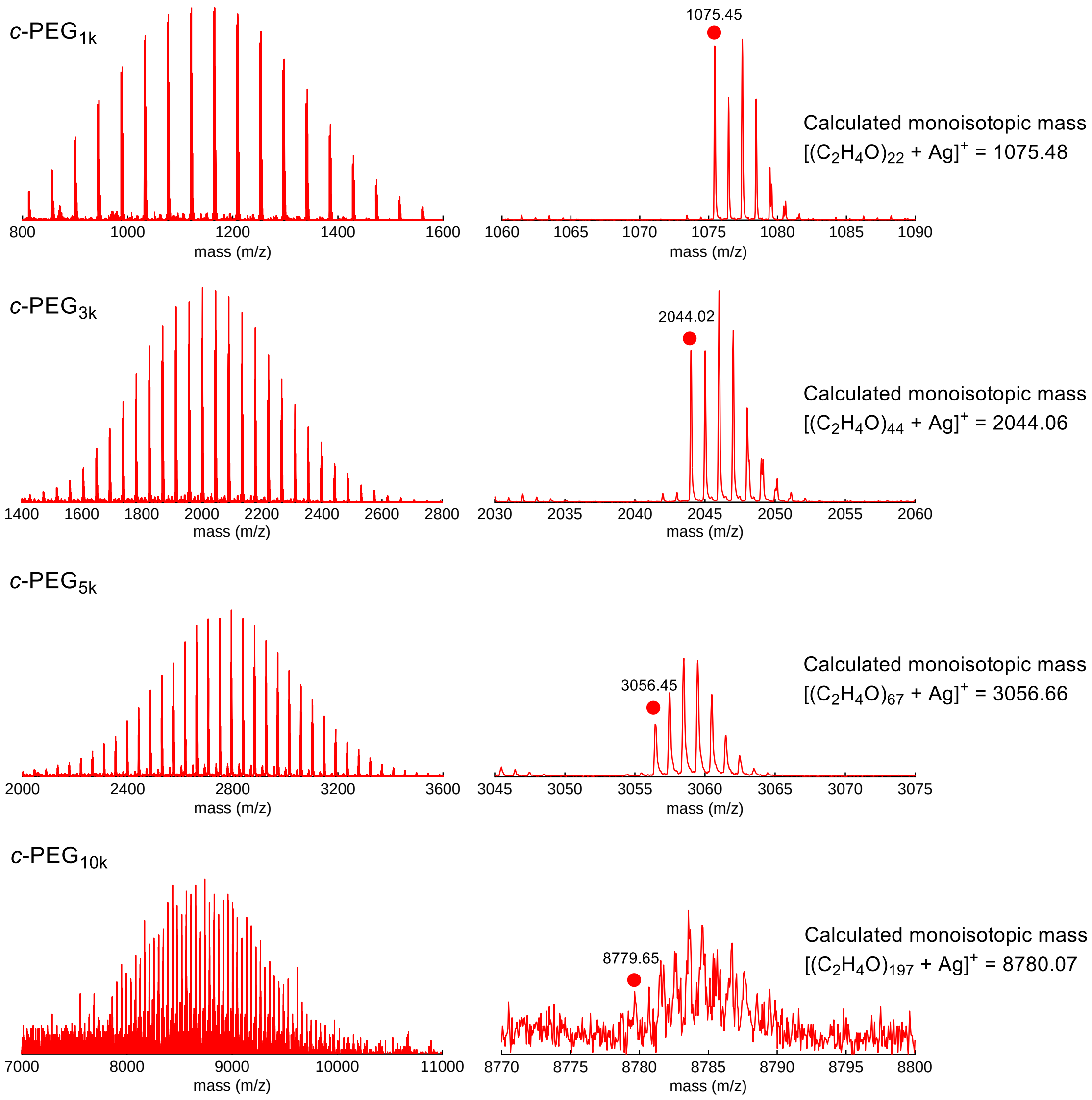


**Supplementary Fig. 3 MALDI-TOF mass spectra of *c*-PEG$_{1k}$, *c*-PEG$_{3k}$, *c*-PEG$_{5k}$, and *c*-PEG$_{10k}$.** Calculated monoisotopic mass and experimental *m*/*z* values of typical peaks are shown.

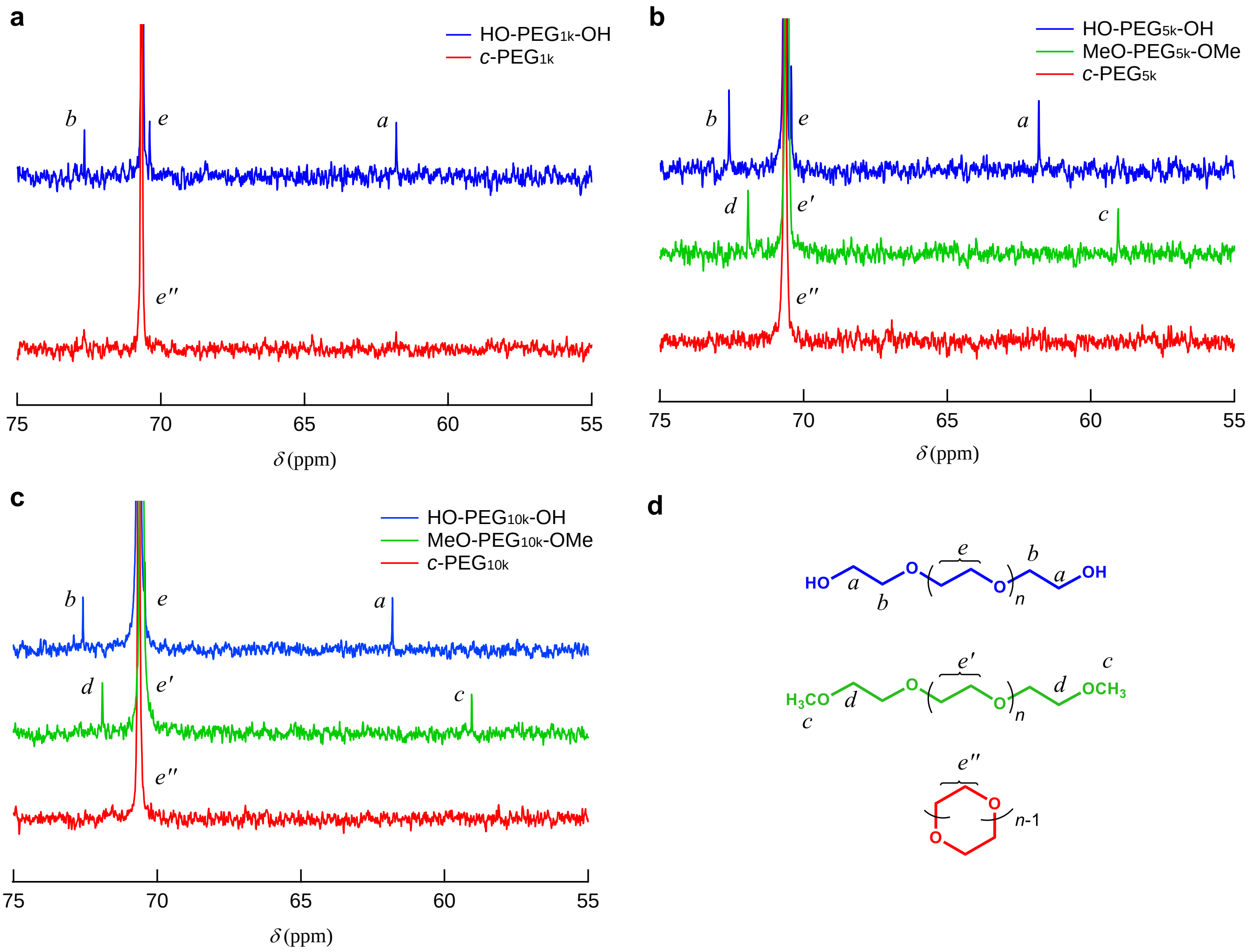


**Supplementary Fig. 4 $^{13}$C NMR spectra and chemical structures of HO–PEG–OH (blue), MeO–PEG–OMe (green), and *c*-PEG (red).** The molecular weight was **a** 1, **b** 5, and **c** 10 kDa. The assigned peaks correspond to the chemical structures in **d**.

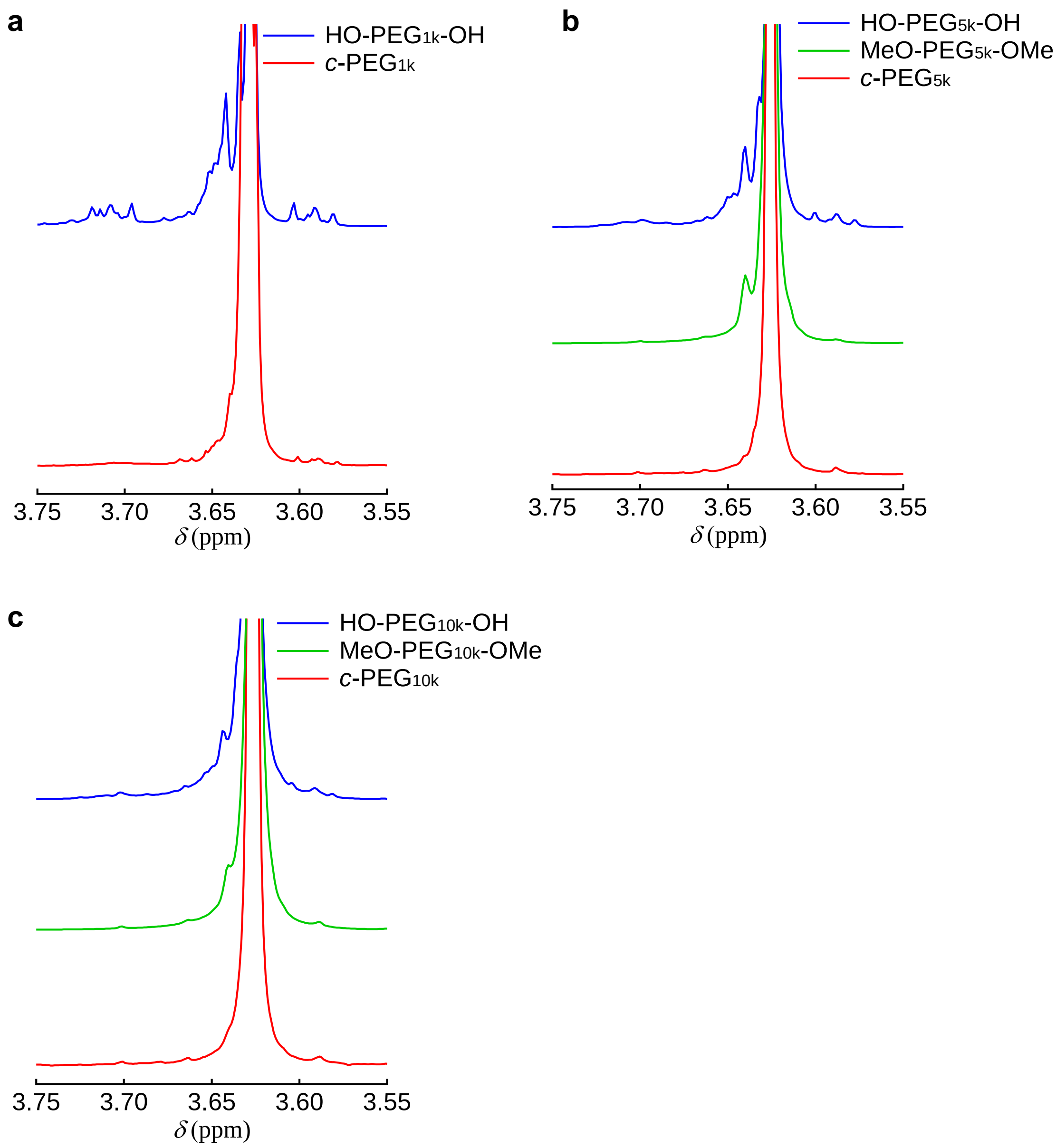


**Supplementary Fig. 5 $^1$H NMR spectra of HO–PEG–OH (blue), MeO–PEG–OMe (green), and *c*-PEG (red).** The molecular weight was **a** 1, **b** 5, and **c** 10 kDa.

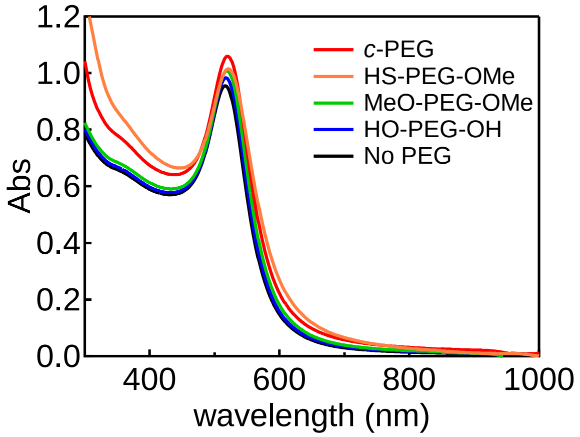


**Supplementary Fig. 6 UV–Vis spectra before freezing and lyophilization.** $AuNPs_{15}$/No PEG (black), $AuNPs_{15}$/HO–$PEG_{3k}$–OH (blue), $AuNPs_{15}$/MeO–$PEG_{3k}$–OMe (green), $AuNPs_{15}$/HS–$PEG_{3k}$–OMe (orange), and $AuNPs_{15}$/*c*-$PEG_{3k}$ (red).

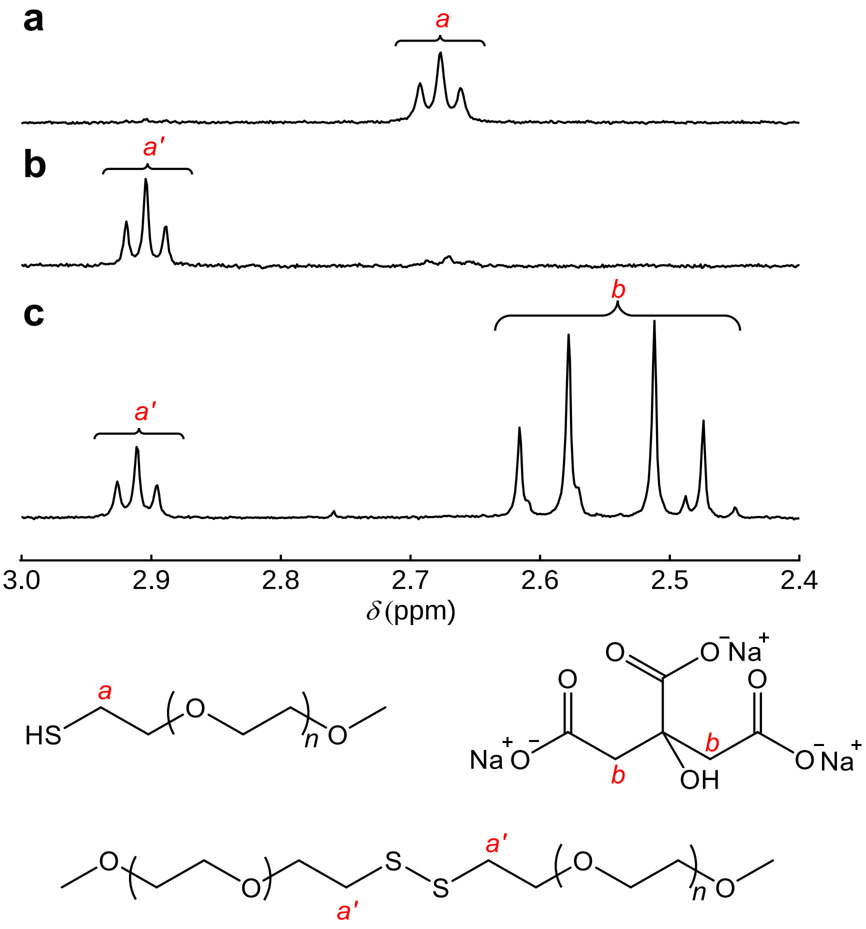


**Supplementary Fig. 7 Formation of disulfide by heating.** $^{1}$H NMR spectra of **a** HS–PEG$_{3k}$–OMe, **b** MeO–PEG$_{3k}$–S–S–PEG$_{3k}$–OMe, and **c** AuNPs$_{15}$/HS–PEG$_{3k}$–OMe after heating at 85 °C for 4 h followed by exchange of the solvent to $D_2O$.

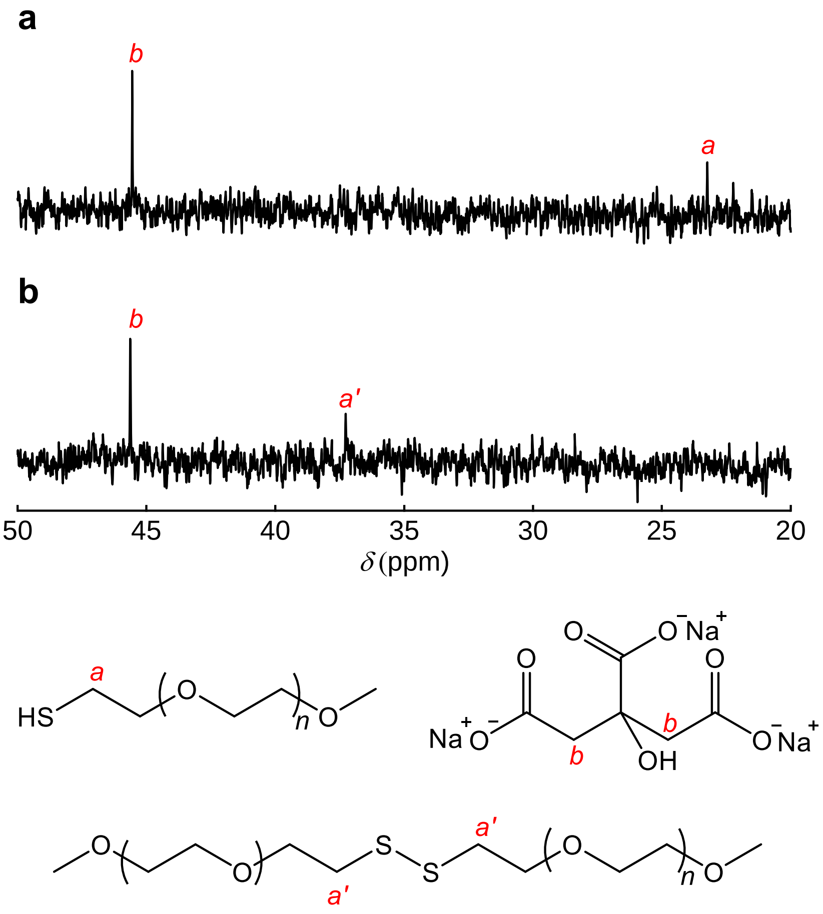


**Supplementary Fig. 8 Formation of disulfide by heating.** $^{13}$C NMR spectra of AuNPs$_{15}$/HS–PEG$_{3k}$–OMe **a** before and **b** after heating at 85 °C for 4 h.

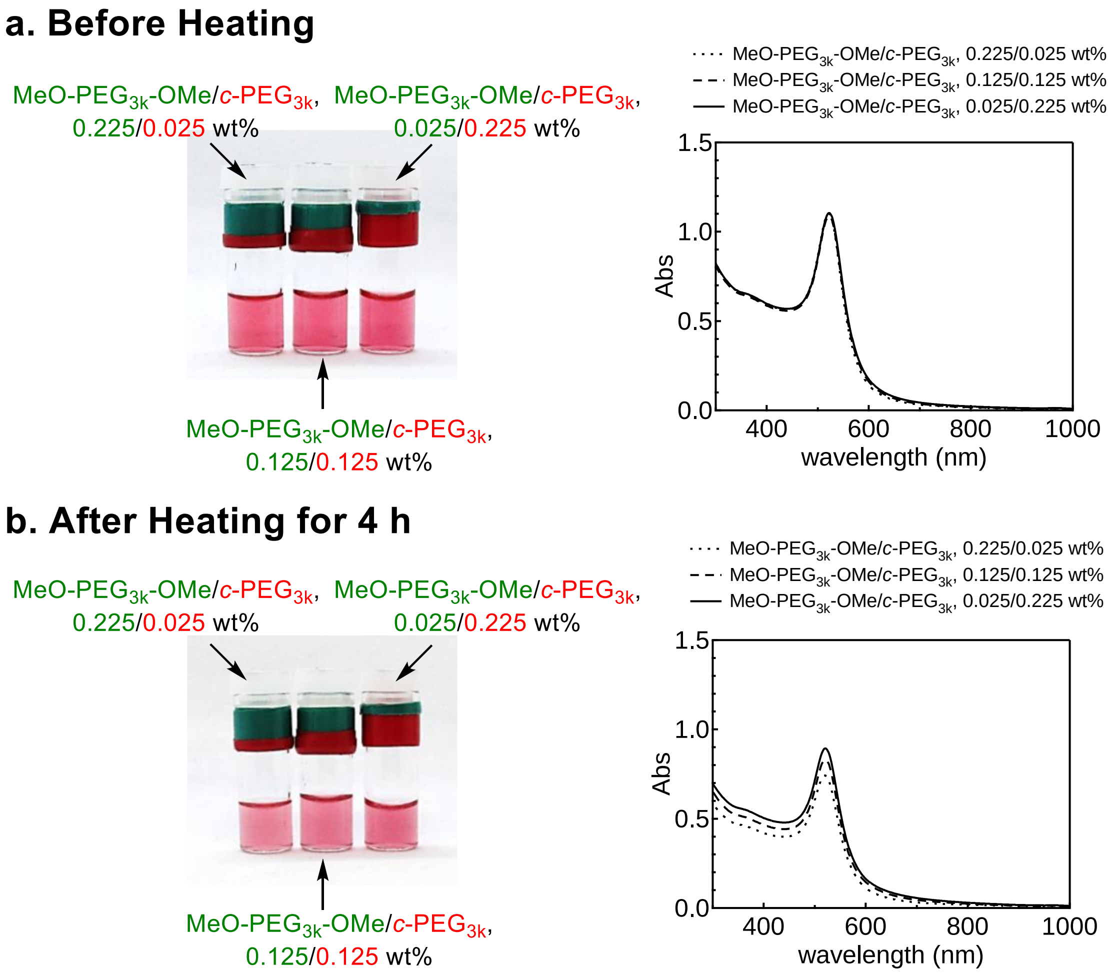


**Supplementary Fig. 9 Heating test.** Photographs and UV–Vis spectra of $AuNPs_{30}$/MeO–$PEG_{3k}$–OMe/*c*-$PEG_{3k}$. **a** Before heating and **b** after heating for 4 h at 85 °C. From left to right in each photograph, the concentrations of MeO–$PEG_{3k}$–OMe/*c*-$PEG_{3k}$ were 0.225/0.025, 0.125/0.125, and 0.025/0.225 wt%. Rel. Abs was 67, 76, or 81%, respectively.

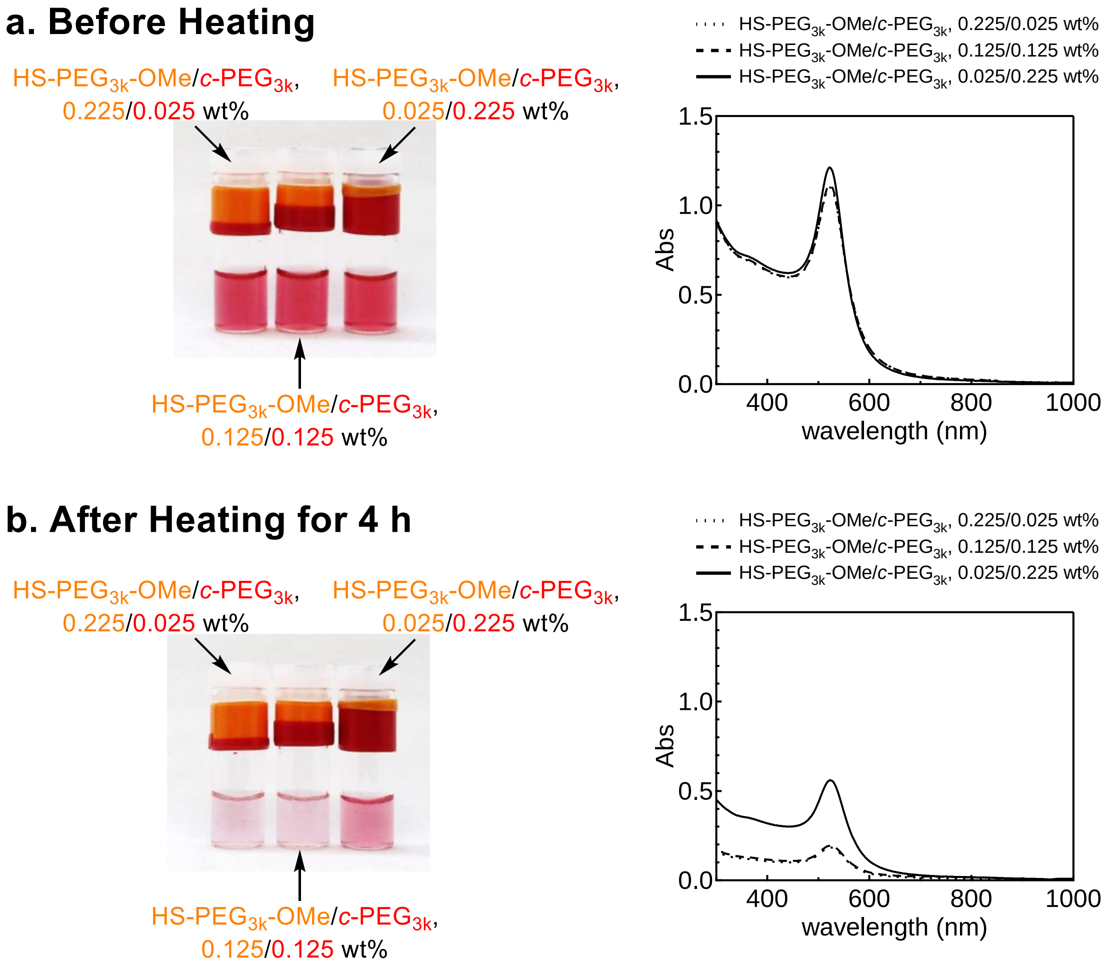


**Supplementary Fig. 10 Heating test.** Photographs and UV–Vis spectra of $AuNPs_{30}$/HS–$PEG_{3k}$–OMe/*c*-$PEG_{3k}$. **a** Before heating and **b** after heating for 4 h at 85 °C. From left to right in each photograph, the concentrations of HS–$PEG_{3k}$–OMe/*c*-$PEG_{3k}$ were 0.225/0.025, 0.125/0.125, and 0.025/0.225 wt%. Rel. Abs was 17, 17, or 46%, respectively.

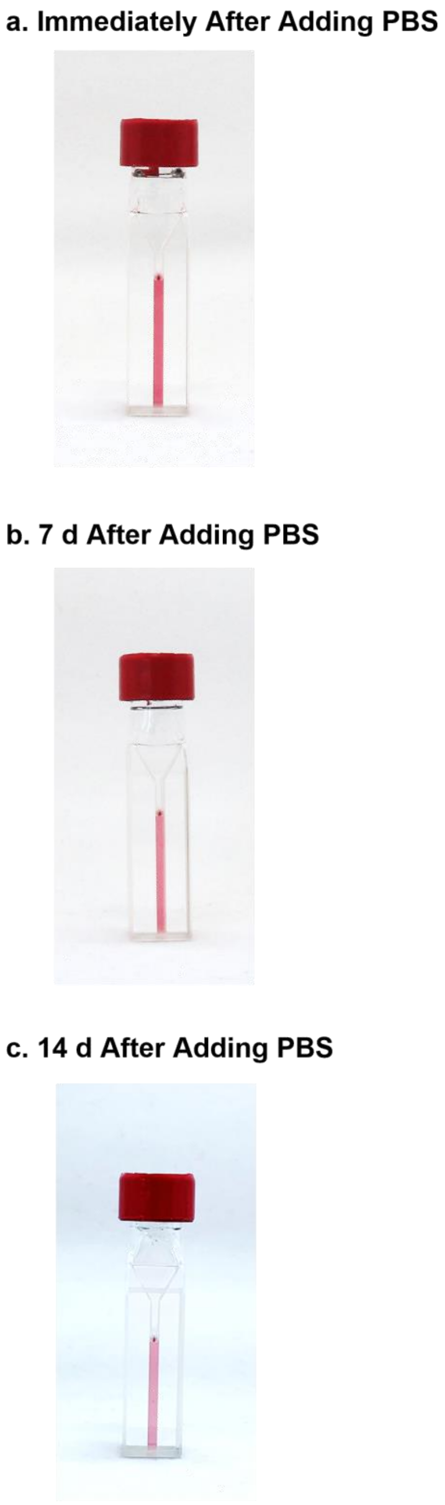


**Supplementary Fig. 11 Photographs of $AuNPs_{15}$/*c*-$PEG_{3k}$ in a long-term stability test. a** Immediately after, **b** 7 d after, and **c** 14 d after the addition of a tenfold-concentrated PBS solution kept at 37 °C.

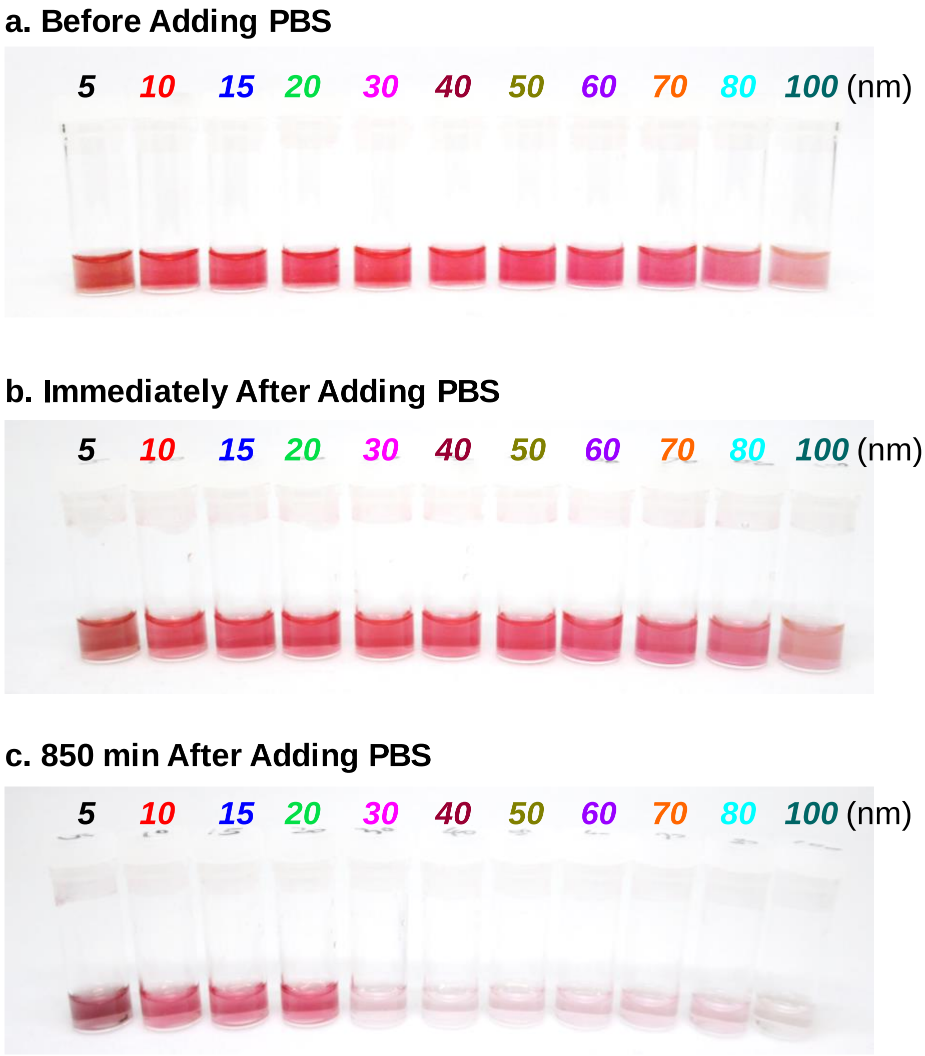


**Supplementary Fig. 12 Physiological condition test with various diameters of AuNPs (5–100 nm).** Photographs of AuNPs/*c*-$PEG_{3k}$ **a** before, **b** immediately after, and **c** 850 min after the addition of a tenfold-concentrated PBS solution. The resulting dispersions were pH 7.4 and 150 mM of NaCl. From left to right in each photograph, $AuNPs_{5}$/*c*-$PEG_{3k}$, $AuNPs_{10}$/*c*-$PEG_{3k}$, $AuNPs_{15}$/*c*-$PEG_{3k}$, $AuNPs_{20}$/*c*-$PEG_{3k}$, $AuNPs_{30}$/*c*-$PEG_{3k}$, $AuNPs_{40}$/*c*-$PEG_{3k}$, $AuNPs_{50}$/*c*-$PEG_{3k}$, $AuNPs_{60}$/*c*-$PEG_{3k}$, $AuNPs_{70}$/*c*-$PEG_{3k}$, $AuNPs_{80}$/*c*-$PEG_{3k}$, and $AuNPs_{100}$/*c*-$PEG_{3k}$.

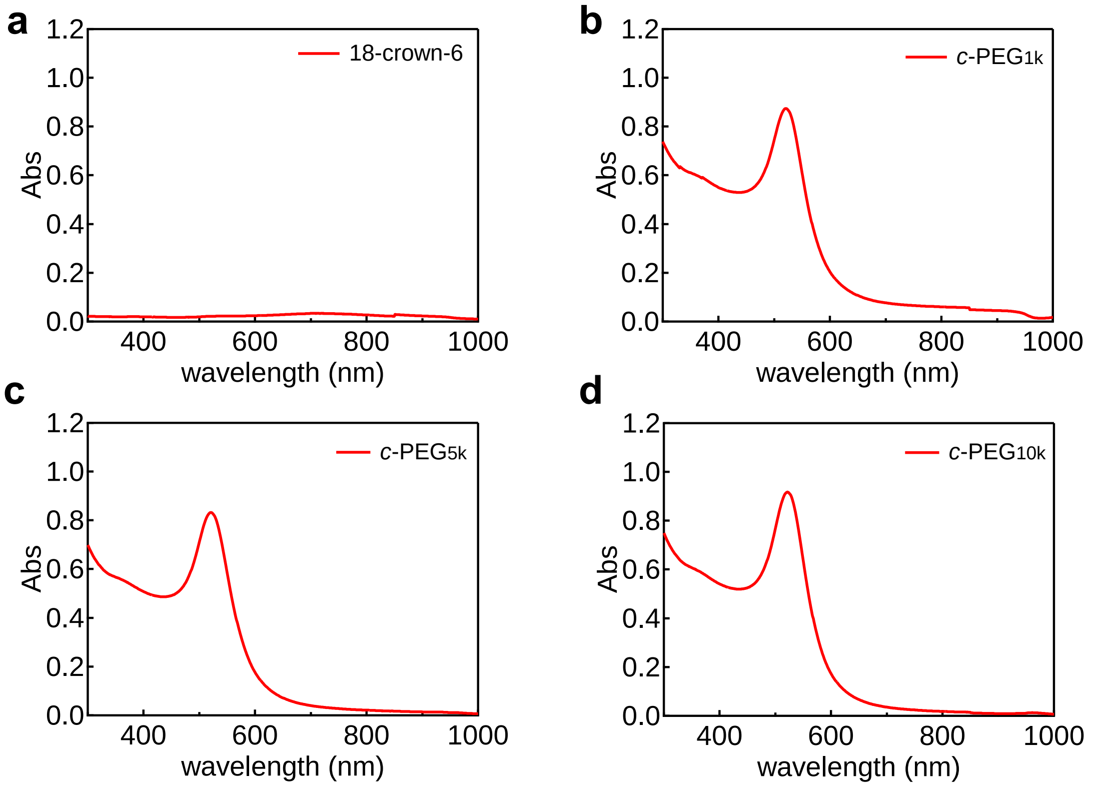


**Supplementary Fig. 13 Physiological condition test of AuNPs/18-crown-6 and AuNPs/*c*-PEG with various sizes.** UV–Vis spectra of **a** $AuNPs_{15}$/18-crown-6, **b** $AuNPs_{15}$/*c*-$PEG_{1k}$, **c** $AuNPs_{15}$/*c*-$PEG_{5k}$, and **d** $AuNPs_{15}$/*c*-$PEG_{10k}$ 1000 min after the addition of a tenfold-concentrated PBS solution. The resulting dispersions were pH 7.4 and 150 mM of NaCl.

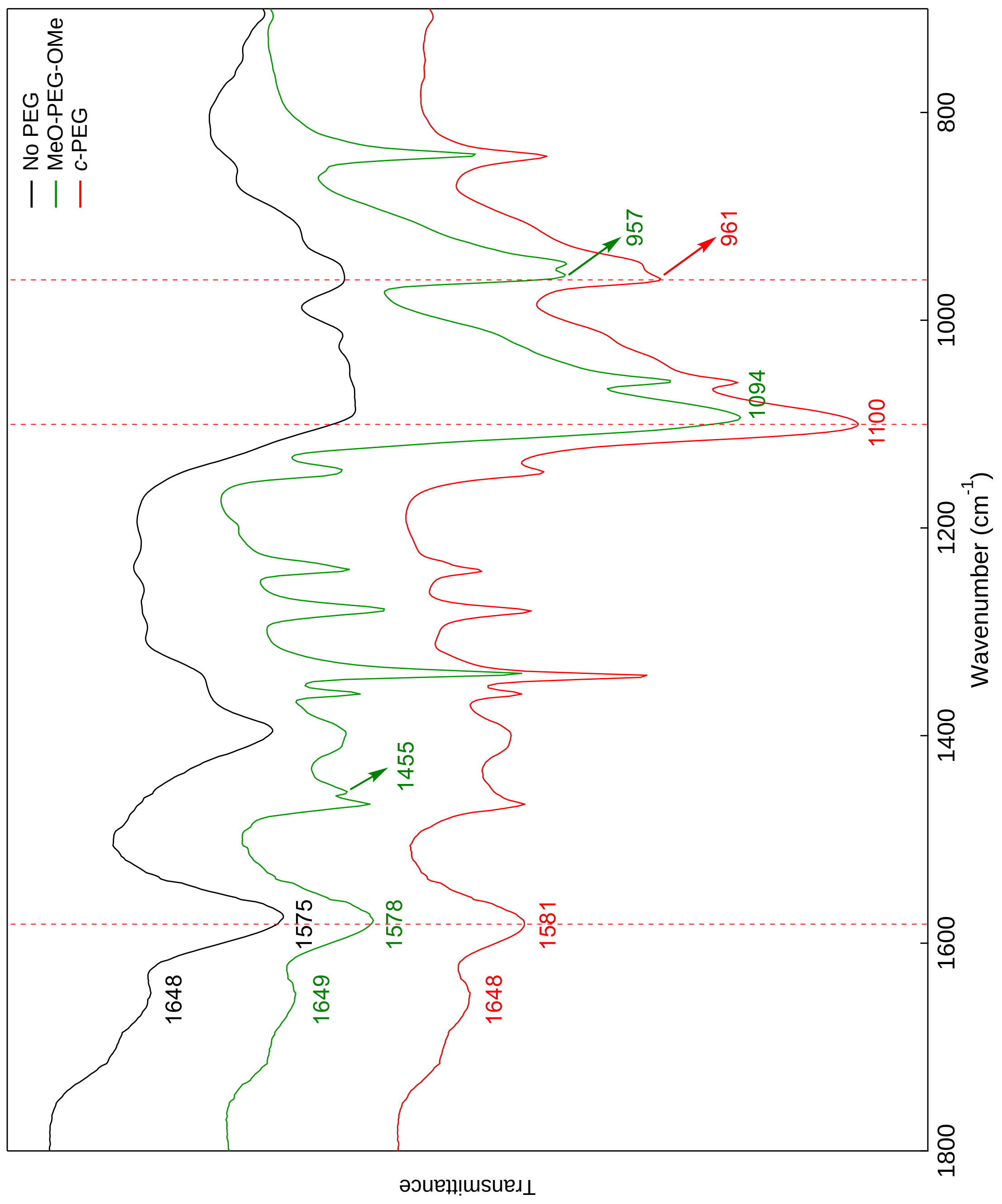


**Supplementary Fig. 14 FT-IR spectra of AuNPs$_{15}$/No PEG (black), AuNPs$_{15}$/MeO–PEG$_{3k}$–OMe (green), and AuNPs$_{15}$/*c*-PEG$_{3k}$ (red).** Red dotted lines indicate the peak top wavenumbers of AuNPs$_{15}$/*c*-PEG$_{3k}$.

**Supplementary Table 1 Experimental and calculated *m*/*z* values for the monoisotopic mass of *c*-$PEG_{3k}$.**

| $DP_n$ | Experimental | Calculated |
|---|---|---|
| 33 | 1559.73 | 1559.77 |
| 34 | 1603.76 | 1603.80 |
| 35 | 1647.80 | 1647.82 |
| 36 | 1691.81 | 1691.85 |
| 37 | 1735.83 | 1735.86 |
| 38 | 1779.86 | 1779.90 |
| 39 | 1823.89 | 1823.93 |
| 40 | 1867.90 | 1867.95 |
| 41 | 1911.94 | 1911.98 |
| 42 | 1955.96 | 1956.01 |
| 43 | 1999.98 | 2000.03 |
| 44 | 2044.02 | 2044.06 |
| 45 | 2088.03 | 2088.08 |
| 46 | 2132.06 | 2132.11 |
| 47 | 2176.07 | 2176.14 |
| 48 | 2220.10 | 2220.16 |
| 49 | 2264.13 | 2264.19 |
| 50 | 2308.16 | 2308.22 |
| 51 | 2352.17 | 2352.24 |
| 52 | 2396.20 | 2396.27 |
| 53 | 2440.22 | 2440.29 |
| 54 | 2484.25 | 2484.32 |
| 55 | 2528.27 | 2528.35 |

**Supplementary Table 2 DLS size, *c*-PEG layer thickness, and grafting density ($\sigma$) of $AuNPs_{15}$/*c*-$PEG_{3k}$ at various concentrations of *c*-$PEG_{3k}$.**

| *c*-PEG conc. (wt%) | 0.05 | 0.15 | 0.25 | 0.50 | 0.75 |
|---|---|---|---|---|---|
| DLS size (nm) | 20.3 | 25.0 | 27.4 | 27.1 | 28.7 |
| *c*-PEG layer thickness (nm) | 1.0 | 3.3 | 4.6 | 4.4 | 5.2 |
| $\sigma$ (chains/nm$^2$) | 0.029 | 0.094 | 0.13 | 0.12 | 0.15 |

**Supplementary Table 3 Estimated numbers of Au atoms and PEG molecules per one AuNP in the case of a PEG concertation of 0.25 wt%.**

| AuNPs size (nm) | Number of Au atoms per one AuNP ($10^4$) | Number of PEG ($M_n$ = 3,000 Da) per one AuNPs ($10^4$) |
|---|---|---|
| 5 | 0.39 | 1.41 |
| 10 | 3.10 | 11.3 |
| 15 | 10.5 | 38.2 |
| 20 | 24.8 | 90.4 |
| 30 | 83.3 | 303 |
| 40 | 198 | 723 |
| 50 | 386 | 1410 |
| 60 | 665 | 2430 |
| 70 | 1070 | 3880 |
| 80 | 1580 | 5780 |
| 100 | 3090 | 11300 |

**Supplementary Table 4 Absorption wavenumbers in the FT-IR spectra of $AuNPs_{15}$/No PEG, $AuNPs_{15}$/MeO–$PEG_{3k}$–OMe, and $AuNPs_{15}$/*c*-$PEG_{3k}$.**

| | AuNPs/No PEG ($cm^{-1}$) | AuNPs/MeO–PEG–OMe ($cm^{-1}$) | AuNPs/*c*-PEG ($cm^{-1}$) |
|---|---|---|---|
| C–O, C–C stretching, $CH_2$ rocking of PEG[1] | – | 841 | 842 |
| $CH_2$ rocking, $CH_2$ twisting of PEG[1] | – | 946 | 946 |
| **$CH_2$ rocking, $CH_2$ twisting of PEG[1]** | **–** | **957** | **961** |
| C–O, C–C stretching, $CH_2$ rocking of PEG[1] | – | 1059 | 1060 |
| **C–O, C–C stretching of PEG[1]** | **–** | **1094** | **1100** |
| C–O stretching, $CH_2$ rocking of PEG[1] | – | 1145 | 1146 |
| $CH_2$ twisting of PEG[1] | – | 1240 | 1242 |
| $CH_2$ twisting of PEG[1] | – | 1279 | 1280 |
| $CH_2$ wagging of PEG[1] | – | 1340 | 1343 |
| Symmetric stretching of $COO^-$ of sodium citrate[2] | 1396 | 1398 | 1399 |
| **$CH_2$ scissoring of PEG[1]** | **–** | **1455** | **–** |
| $CH_2$ scissoring of PEG[1] | – | 1466 | 1466 |
| **Asymmetric stretching of $COO^-$ of sodium citrate[2]** | **1575** | **1578** | **1581** |
| Asymmetric stretching of $COO^-$ of Au–citrate[2] | 1648 | 1649 | 1648 |

Noticeable differences are shown in bold.

## Supplementary references

1. Vrandečić, N.S., Erceg, M., Jakić, M. & Klarić, I. Kinetic analysis of thermal degradation of poly(ethylene glycol) and poly(ethylene oxide)s of different molecular weight. *Thermochim. Acta* **498**, 71-80 (2010).

2. Wulandari, P. et al. Characterization of citrates on gold and silver nanoparticles. *J. Colloid Interface Sci.* **438**, 244-248 (2015).
