# Supplementary material for: Enhanced dispersion stability of gold nanoparticles by the physisorption of cyclic poly(ethylene glycol)
Source: Nat Commun. 2020 Nov 30;11:6089. doi: 10.1038/s41467-020-19947-8 (PMC7705015; doi:10.1038/s41467-020-19947-8)
Supplement: Supplementary file 2 — Reporting Summary [file 41467_2020_19947_MOESM2_ESM.pdf]

nature research

Corresponding author(s): Takuya Yamamoto

Last updated by author(s): Oct 5, 2020

Reporting Summary

Nature Research wishes to improve the reproducibility of the work that we publish. This form provides structure for consistency and transparency in reporting. For further information on Nature Research policies, see our Editorial Policies and the Editorial Policy Checklist.

Statistics

For all statistical analyses, confirm that the following items are present in the figure legend, table legend, main text, or Methods section.

n/a	Confirmed	
☐	☒	The exact sample size (n) for each experimental group/condition, given as a discrete number and unit of measurement
☐	☒	A statement on whether measurements were taken from distinct samples or whether the same sample was measured repeatedly
☒	☐	The statistical test(s) used AND whether they are one- or two-sided<br>*Only common tests should be described solely by name; describe more complex techniques in the Methods section.*
☒	☐	A description of all covariates tested
☒	☐	A description of any assumptions or corrections, such as tests of normality and adjustment for multiple comparisons
☐	☒	A full description of the statistical parameters including central tendency (e.g. means) or other basic estimates (e.g. regression coefficient) AND variation (e.g. standard deviation) or associated estimates of uncertainty (e.g. confidence intervals)
☒	☐	For null hypothesis testing, the test statistic (e.g. F, t, r) with confidence intervals, effect sizes, degrees of freedom and P value noted<br>*Give P values as exact values whenever suitable.*
☒	☐	For Bayesian analysis, information on the choice of priors and Markov chain Monte Carlo settings
☒	☐	For hierarchical and complex designs, identification of the appropriate level for tests and full reporting of outcomes
☒	☐	Estimates of effect sizes (e.g. Cohen's d, Pearson's r), indicating how they were calculated

Our web collection on statistics for biologists contains articles on many of the points above.

Software and code

Policy information about availability of computer code

Data collection	No software was used.
Data analysis	No software was used.

For manuscripts utilizing custom algorithms or software that are central to the research but not yet described in published literature, software must be made available to editors and reviewers. We strongly encourage code deposition in a community repository (e.g. GitHub). See the Nature Research guidelines for submitting code & software for further information.

Data

Policy information about availability of data

All manuscripts must include a data availability statement. This statement should provide the following information, where applicable:

- Accession codes, unique identifiers, or web links for publicly available datasets
- A list of figures that have associated raw data
- A description of any restrictions on data availability

The data that support the findings of this study are available from the corresponding authors upon reasonable request. Source data are provided with this paper.

Field-specific reporting

Life sciences study design

All studies must disclose on these points even when the disclosure is negative.

Sample size	In all experiments, the sample sizes were determined by estimating the minimum number of samples to obtain a statistically significant difference. Tumor growth was measured by caliper measurement and tumor volumes were calculated by the following equation: V = (a × b^2)/2, where a refers to the tumor width (mm) and b refers to the tumor length (mm).
Data exclusions	In antitumour experiments (Figure 5e and 5f), the data that were more largely deviated from the average value (50 mm^3) were excluded to make the sample size uniform. For example, due to the inoculataion mistake, mice having over 200 mm^3 tumor (one mouse) and 0 mm^3 tumor (one mouse) were excluded before staring the experiments.
Replication	We performed our biodistribution study as major experiments. Those were performed once because we obtain statistical significances (p<0.05) by student t-test.
Randomization	In in vivo experiments using tumor-bearing mice, mice were randomly allocated to each group to have the similar tumour size in average.
Blinding	The investigators who measure Au concentration in blood and tumor were blinded for data collection to provide impartial judgment.

Reporting for specific materials, systems and methods

We require information from authors about some types of materials, experimental systems and methods used in many studies. Here, indicate whether each material, system or method listed is relevant to your study. If you are not sure if a list item applies to your research, read the appropriate section before selecting a response.

Materials & experimental systems

n/a	Involved in the study	
☒	☐	Antibodies
☐	☒	Eukaryotic cell lines
☒	☐	Palaeontology and archaeology
☐	☒	Animals and other organisms
☒	☐	Human research participants
☒	☐	Clinical data
☒	☐	Dual use research of concern

Methods

n/a	Involved in the study	
☒	☐	ChIP-seq
☒	☐	Flow cytometry
☒	☐	MRI-based neuroimaging

Eukaryotic cell lines

Policy information about cell lines

Cell line source(s)	Murine colon adenocarcinoma (C26) was supplied from the National Cancer Center, Tokyo, Japan
Authentication	N/A
Mycoplasma contamination	No mycoplasms were detected in our cell lines.
Commonly misidentified lines (See ICLAC register)	No commonly misindentified cell lines were used.

Animals and other organisms

Policy information about studies involving animals; ARRIVE guidelines recommended for reporting animal research

Laboratory animals	BALB/c mice (6-week-old female) were used for blood circulation test and preparation of a subcutaneous C26 tumour model . Animals were kept in a temperature-controlled room on 12 h / 12 h light / dark schedule at 22 degC with food and water ad libitum.
Wild animals	No wild animals were used in this study.
Field-collected samples	No field-collected samples were used in this study.
Ethics oversight	Animal experiments using subcutaneous tumour-bearing mice were performed in accordance with the guidelines for animal experiments at The University of Tokyo, Tokyo Institute of Technology, and Hokkaido University, Japan.

Note that full information on the approval of the study protocol must also be provided in the manuscript.
